# Reversible Co(II)–Co(III) Transformation in a Family of Metal–Dipyrazolate Frameworks

**DOI:** 10.1021/jacs.4c09173

**Published:** 2024-10-08

**Authors:** Xiang-Jing Kong, Tao He, Andrey A. Bezrukov, Shaza Darwish, Guang-Rui Si, Yong-Zheng Zhang, Wei Wu, Yingjie Wang, Xia Li, Naveen Kumar, Jian-Rong Li, Michael J. Zaworotko

**Affiliations:** † Beijing Key Laboratory for Green Catalysis and Separation and Department of Chemical Engineering, College of Materials Science and Engineering, Beijing University of Technology, Beijing 100124, PR China; ‡ Bernal Institute and Department of Chemical Sciences, University of Limerick, Limerick V94 T9PX, Ireland; § Shandong Provincial Key Laboratory of Monocrystalline Silicon Semiconductor Materials and Technology, College of Chemistry and Chemical Engineering, Dezhou University, Dezhou 253023, China

## Abstract

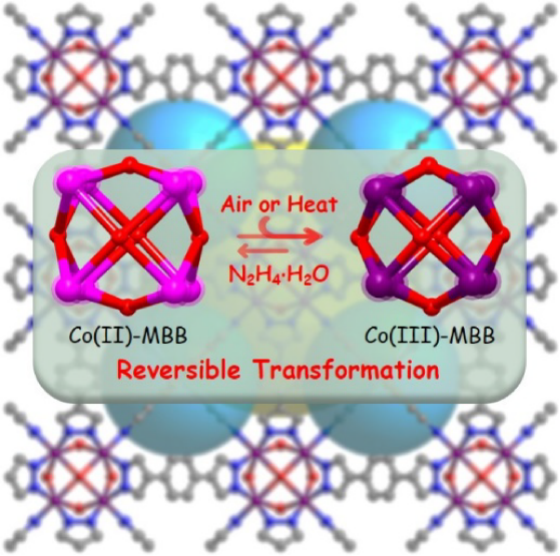

Transformation between oxidation states is widespread in transition metal coordination chemistry and biochemistry, typically occurring in solution. However, air-induced oxidation in porous crystalline solids with retention of crystallinity is rare due to the dearth of materials with high structural stability that are inherently redox active. Herein, we report a new family of such materials, four isostructural cobalt–pyrazolate frameworks of face-centered cubic, **fcu**, topology, **fcu-L-Co**, that are sustained by Co_8_ molecular building blocks (MBBs) and dipyrazolate ligands, **L**. **fcu-L-Co** were observed to spontaneously transform from Co(II)_8_ to Co(III)_8_ MBBs in air with retention of crystallinity, marking the first such instance in metal–organic frameworks (MOFs). This transformation can also be achieved through water vapor sorption cycling, heating, or chemical oxidation. The reverse reactions were conducted by exposure of **fcu-L-Co(III)** to aqueous hydrazine. **fcu-L-Co(II)** exhibited high gravimetric water vapor uptakes of 0.55–0.68 g g^–1^ at 30% relative humidity (RH), while in **fcu-L-Co(III)** the inflection point shifted to lower RH and framework stability improved. Insight into the transformation between **fcu-L-Co(II)** and **fcu-L-Co(III)** was gained from single crystal X-ray diffraction and *in situ* spectroscopy. Overall, the crystal engineering approach we adopted has afforded a new family of MOFs that exhibit cobalt redox chemistry in a confined space coupled with high porosity.

## Introduction

That transition metals can exist in multiple oxidation states, which is thanks to the existence of partially filled *d* orbitals, means that they can exhibit useful magnetic, optical, and redox properties.^1,2^ The ability to transform between different oxidation states through electron transfer enables coordination complexes to participate in various chemical reactions and life processes,^3−5^ as exemplified by the redox chemistry of the prototypal Werner complex [Co(NH_3_)_6_]^2+^, which can transform to [Co(NH_3_)_6_]^3+^ in air,^6^ and the conversion between Fe(II) and Fe(III) in hemoglobin and myoglobin, which enables oxygen transportation and supply in cells and tissues.^7^ Similarly, the oxidation state of cobalt is crucial for the biological activity of vitamin B12, where Co(III) is usually involved in enzymatic reactions and certain metabolic processes.^8^ Although the existence of multiple oxidation states in transition metals is so fundamental in chemistry and biochemistry, such redox chemistry in porous crystalline materials such as metal–organic frameworks^9^ (MOFs) and porous coordination networks (PCNs)^10^ remains understudied.

MOFs and PCNs have attracted growing interest for their promise in a diverse range of applications.^11,12^ Transition metals are extensively used in constructing MOFs due to their ability to form molecular building blocks (MBBs)^13^ with predictable coordination geometry and connectivity as well as the properties that they bring, including for catalysis.^2,9,14,15^ The oxidation state of a transition metal MBBs can profoundly impact structural features and gas adsorption performance.^16,17^ Redox activity has been demonstrated in MBB-based MOFs containing Cr_2_(COO)_4_ [Cr(II)/Cr(III)], M_3_(μ_3_-O)(COO)_6_ [*M* = Cr(II)/Cr(III), Fe(II)/Fe(III), and Co(II)/Co(III)], Mn_4_Cl(tetrazolate)_8_^–^ [Mn(II)/Mn(III)], Ce_6_O_4_(OH)_4_(COO)_12_ [Ce(III)/Ce(IV)], and Ti_8_O_8_(OH)_4_(COO)_12_ [Ti(III)/Ti(IV)].^15−23^ Among these examples, the complete transformation of nodal oxidation states has rarely been established. Labile Fe(II)- and Cr(II)-MOFs were converted to PCN-426-M(III) analogs by air oxidation.^16^ Reversible cleavage and formation of O=O bonds were achieved on tetramanganese clusters in Mn_3_[(Mn_4_Cl)_3_BTT_8_]_2_ (BTT = 1,3,5-benzene-tristetrazolate), by Mn(II)/Mn(III) redox chemistry.^17^ The previous reports on Co(II)/Co(III) redox mainly include cases of mixed-valent Co(II)/Co(III) materials or examples with transient Co(III) species during catalysis.^24−27^ Complete Co(II)/Co(III) redox is even rare in MOFs. Dinca’s group studied the chemisorption of Cl_2_/Br_2_ in Co_2_Cl_2_BTDD, leading to Co_2_Cl_2_*X*_2_BTDD (*X* = Cl/Br).^28^ Sustained with rod building blocks, RBBs, Co_2_Cl_2_*X*_2_BTDD represents the first Co(III) MOF. To our knowledge, spontaneous and complete Co(II)/Co(III) redox chemistry of metal cluster MBB nodes has not yet been reported in MOFs with structural characterization of multiple redox states, a matter that we address herein.

**Figure 1:**
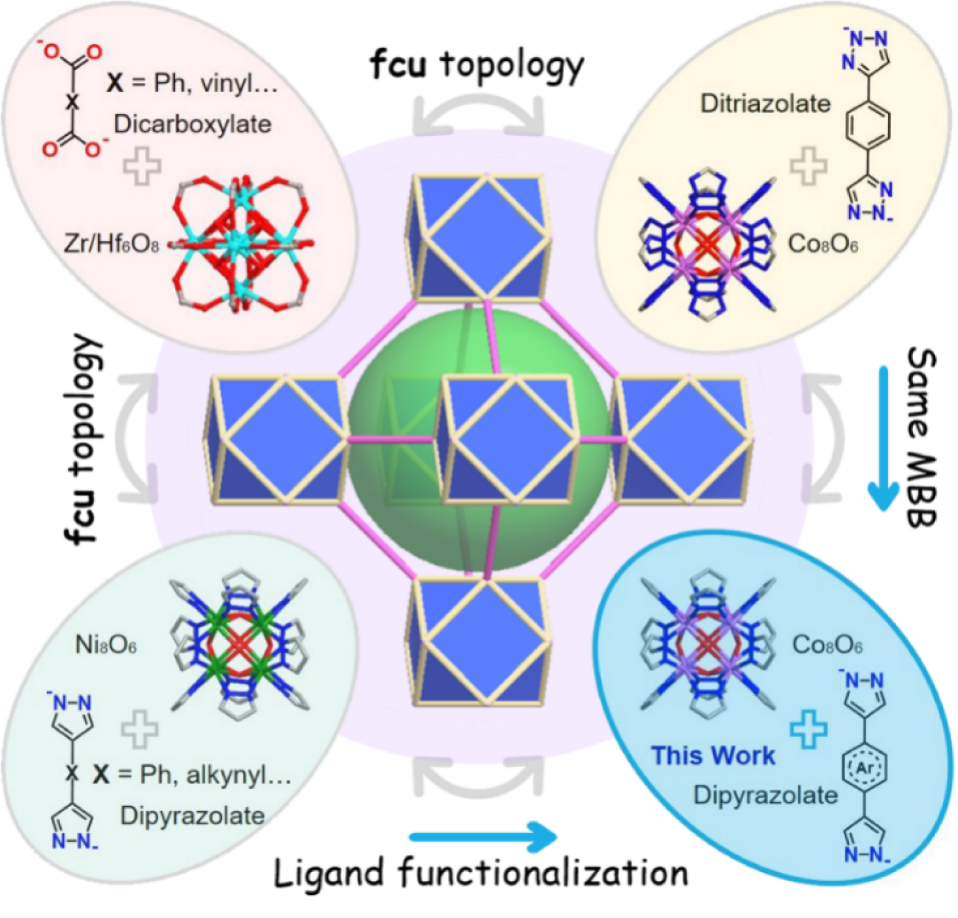
Schematic representation of the crystal engineering approach used herein to access new **fcu** MOFs, **fcu-L-Co**, by combining Co_8_ MBBs and dipyrazolate linkers, highlighting the relationship with existing families of **fcu** MOFs based on 12-connected metal cluster nodes.

Structural stability of MOFs and PCNs is a requirement for their potential utility in gas/vapor sorption and heterogeneous catalysis.^29−32^**fcu** topology has been targeted for design of stable MOFs thanks to the availability of high connectivity nodes such as 12-connected [Zr_6_O_4_(OH)_4_(COO)_12_], Zr_6_, and [Ni_8_(OH)_4_(H_2_O)_2_(az)_12_] (az = pyrazolate or triazolate), Ni_8_, and di-topic linker ligands such as dicarboxylates and diazolates (Figure 1).^33−35^ Zr_6_ MOFs are particularly well-studied,^36^ with MOF-801 and UiO-66 derivatives being stable enough to be of interest for water harvesting applications.^37,38^ Related azolate **fcu** MOFs remain understudied even though they can also offer excellent structural stability under environmental conditions.^27,35,39,40^ Herein, we report the synthesis and properties of a new family of **fcu** MOFs based upon the Co(II)_8_(OH)_6_(pz)_12_^2–^ (pz = pyrazolate) MBB and dipyrazolate linkers (Figure 1). The four isostructural MOFs reported herein, **fcu-L-Co**, [Co_8_(OH)_6_(**L**)_6_·2H_3_O·4H_2_O]_*n*_, exhibit spontaneous (air induced) and reversible transformation from Co(II) to Co(III), and **fcu-L-Co(II)** to **fcu-L-Co(III)**.

## Results and Discussion

### Design and Synthesis

Reports on the crystal engineering synthesis of kinetically stable pyrazolate **fcu**-MOFs have focused on those with Ni-based MBBs. Galli’s and Navarro’s groups employed dipyrazolate linkers to synthesize the [Ni_8_(OH)_4_(H_2_O)_2_(L)_6_]_*n*_ ([Ni_8_(L)_6_], L = dipyrazolate) family.^41,42^ These **fcu** MOFs were studied in terms of gas adsorption and catalysis. Li’s group reported the crystal syntheses and structural properties of BUT-2, [Ni_8_(OH)_4_(H_2_O)_2_(L3)_6_]_*n*_, H_2_L3 = 4,4′-benzene-1,4-diylb-is(1*H*-pyrazole), and its derivatives.^43^**fcu**-MOF analogs based upon Co_8_ MBBs are limited to three reports, two involving triazolate linkers^27,39^ and one with dipyrazolate linkers.^40^ The Co_8_ MBB has also been reported in the **ftw** topology MOF [(NH_4_)_2_·[Co_11_(μ_4_–OH)_6_(CN)_6_(trz)_12_] (trz = 1,2,4-triazolate).^44^

**Figure 2:**
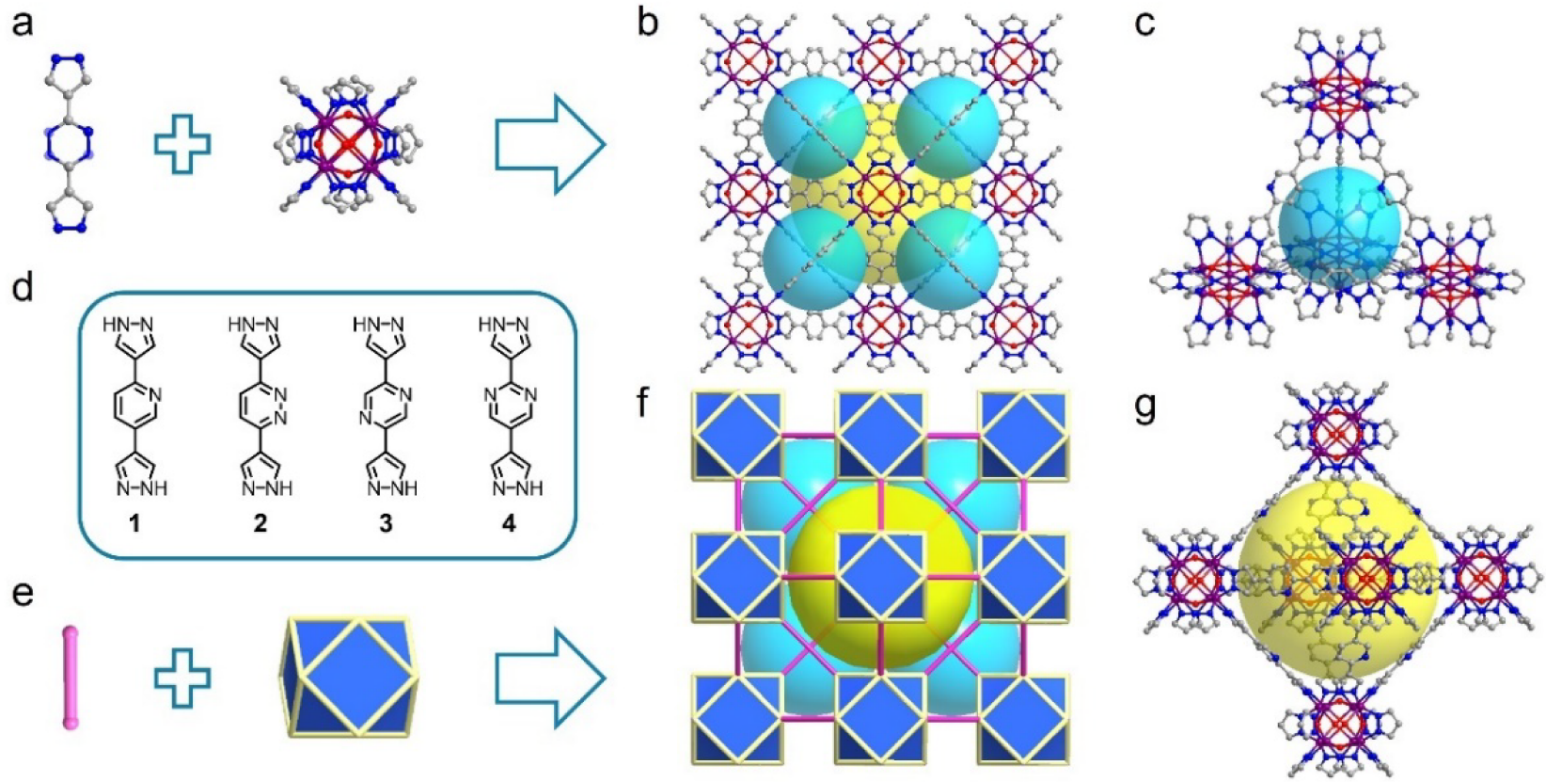
Design and structure of **fcu-L-Co**. (a,d) Dipyrazolate linker ligands **1**–**4** and 12-connected [Co_8_(μ_4_–OH)_6_(pz)_12_]^2–^ MBBs. (b) Structure of **fcu-L-Co** with (c) tetrahedron and (g) octahedron cages. Assembly between (e) linear linkers and icosahedral nodes resulted in (f) **fcu** networks.

We herein report the use of four dipyrazole linker ligands with hydrophilic central aromatic rings (pyridine, pyridazine, pyrazine, and pyrimidine), the recently reported 2,5-di(1*H*-pyrazol-4-yl)pyridine (**1**)^45^ and three new ligands, 3,6-di(1*H*-pyrazol-4-yl)pyridazine (**2**), 2,5-di(1*H*-pyrazol-4-yl)pyrazine (**3**), and 2,5-di(1*H*-pyrazol-4-yl)pyrimidine (**4**) (Figures 2d and S1–S4) to form isostructural **fcu**-MOFs. Solvothermal reactions between **1**–**4** and divalent cobalt salts yielded red/orange octahedral crystals of four new **fcu**-MOFs, **fcu-1-Co(II)**, **fcu-2-Co(II)**, **fcu-3-Co(II)**, and **fcu-4-Co(II)** (Figure 2, see the MOFs Synthesis section in SI for full details). Single crystal X-ray diffraction (SCXRD) analysis revealed that these MOFs are isostructural and sustained by the same [Co_8_(μ_4_–OH)_6_(pz)_12_]^2–^ (Co_8_) MBB.

### Structure and Porosity Characterization

Unit cell and structure refinement parameters for the as-synthesized **fcu-L-Co(II)** MOFs are summarized in Table S1. Using **fcu-3-Co(II)** as an exemplar, it crystallized in the cubic crystal system with space group Fmm. The cobalt atom coordinates to three nitrogen atoms from three pyrazolate moieties and three μ_4_–OH entities in a distorted-octahedral coordination geometry. Eight cobalt atoms are connected by six μ_4_–OH entries to form a Co_8_(μ_4_–OH)_6_ core that can be simplified to a Co_8_ cube, which resembles the Ni_8_ cube found in [Ni_8_(OH)_4_(H_2_O)_2_(L)_6_]_*n*_ and PCN-601.^29,41,42,46^ The Co_8_ cube is linked by 12 pyrazolate moieties from different **3**^2–^ ligands to generate the anticipated **fcu** topology network (Figure 2a). Six H_3_O^+^/H_2_O entities are proximal to the μ_4_–OH moieties (O···O distance 3.894 Å) (Figure S5), two of which are assumed to be H_3_O^+^ cations to balance the overall negative charge of the MBBs (supported by the spectroscopy and thermal analysis results discussed below, and the pH around 7 of the solvothermal system) (Figure S6). The presence of H_3_O^+^ has also been observed in other compounds obtained from DMF.^47,48^ The **3**^2–^ ligand has planar geometry (Figure 2a) to facilitate the **fcu** topology of **fcu-3-Co(II)** (Figure 2a,b,e, and f). Co–N bond distance of 2.0620(23) Å, Co–O bond distance of 2.2076(28) Å, and Co···Co distance of 3.0352(7) Å (Table S2) are consistent with the literature values for Co(II) ions.^27,39,44^ The formula of **fcu-3-Co(II)** is thus assigned as Co(II)_8_(OH)_6_(**3**)_6_(H_3_O^+^)_2_·4H_2_O. As is typical for **fcu** topology, two types of cages are present in **fcu-3-Co(II)**: tetrahedral (sphere of diameter 8.8 Å, without taking van der Waals radii into account) (Figure 2c) and octahedral (17.6 Å diameter) (Figure 2g). These cages connect via hydrophilic windows (sphere of diameter 7.0 Å) enclosed by N-functionalized ligands (Figure S7). The solvent accessible void of **fcu-3-Co(II)** is 61.1% of its unit cell volume as determined by PLATON (Tables S1).^49^

Powder X-ray diffraction (PXRD) analysis was conducted to examine the crystallinity and phase purity of **fcu-L-Co(II)**. The experimental PXRD patterns are consistent with those calculated from single crystal structure data (Figures S8–S11), indicating phase purity. To evaluate porosity, samples were characterized by N_2_ adsorption at 77 K after methanol exchange and evacuation at 80 °C. Saturated N_2_ uptakes of 525, 520, 538, and 567 cm^3^ g^–1^ (STP) were observed for the activated phases of **fcu-1-Co(II)**, **fcu-2-Co(II)**, **fcu-3-Co(II)**, and **fcu-4-Co(II)** (Figure S12), respectively. The Brunauer–Emmett–Teller (BET) surface areas were determined to be 1656, 1642, 1693, and 1750 m^2^ g^–1^, respectively. Thermogravimetric analysis (TGA) revealed that **fcu-1-Co(II)** is stable up to 390 °C, **fcu-2-Co(II)** up to 350 °C, **fcu-3-Co(II)** up to 360 °C, and **fcu-4-Co(II)** up to 400 °C (Figure S13). These materials were also found to exhibit good chemical stability after various treatments (Figures S8–S11 and S14), laying the foundation for utility.

### Structural Transformation

**Figure 3:**
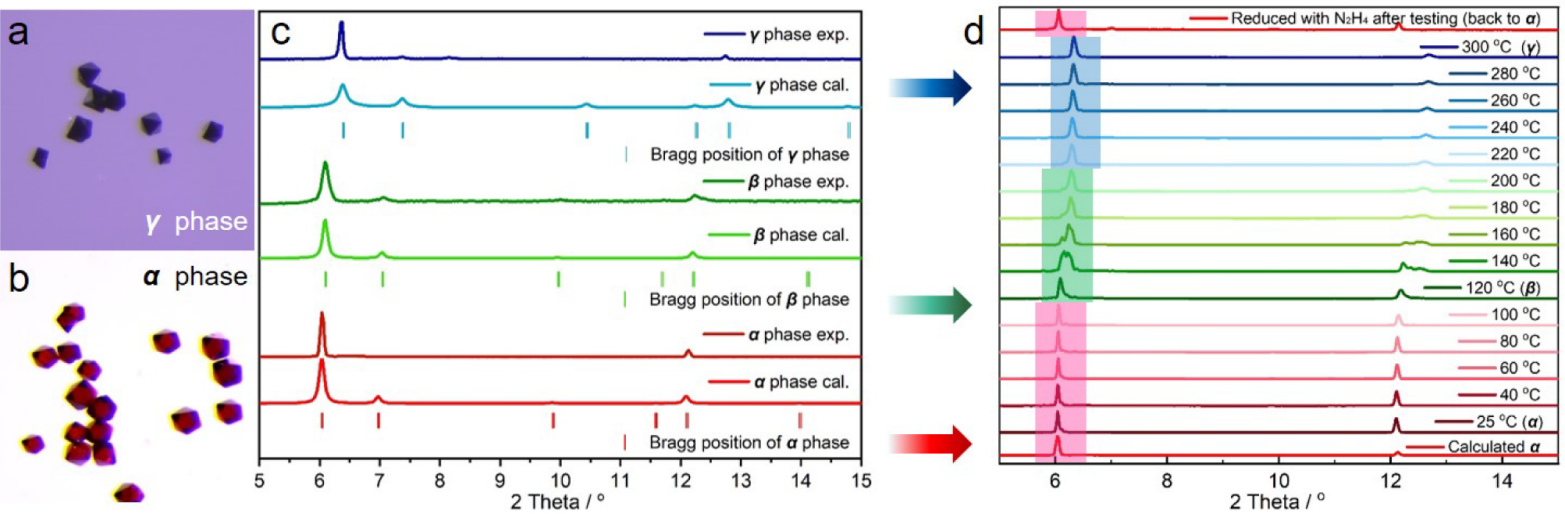
(a,b) Microscopic images and (c) PXRD patterns of different phases of **fcu-3-Co** collected under ambient conditions (as synthesized **fcu-3-Co(II)** (**α** phase), activated **fcu-3-Co(II)** (**β** phase), and oxidized **fcu-3-Co(III)** (**γ** phase)). (d) VT-PXRD patterns of the as-synthesized **fcu-3-Co(II)** (**α** phase) under a vacuum.

Prior to further study, we noted color differences among freshly made (**fcu-L-Co(II)**, **α** phase, red), activated (**fcu-L-Co(II)**, **β** phase, dark red), and long-term air exposed (**fcu-L-Co(III)**, **γ** phase, purple) samples. All of these samples were characterized to explore the relevance between phases (Figures 3 and S15–S31). We take **fcu-3-Co** as an example (Figures 3a–b and S15). **fcu-3-Co(III)** was obtained by exposing MeOH-exchanged **fcu-3-Co(II)** to laboratory air for four months. The characteristic PXRD peaks of **fcu-3-Co(III)** had shifted to higher 2θ values relative to those of **fcu-3-Co(II)** (Figure S14), indicating shrinkage of the crystal structure. Transformation of **fcu-3-Co(II)** also occurred in the presence of aqueous H_2_O_2_/meta-chloroperoxybenzoic acid (*m*-CPBA). **fcu-3-Co(II)** was subsequently recovered by treating **fcu-3-Co(III)** with N_2_H_4_·H_2_O, as indicated by the red color of the resulting crystals and a PXRD pattern matching that of **fcu-3-Co(II)** (Figures S16 and S19). To further study this transformation process, *in situ* variable-temperature(VT)-PXRD experiments under ambient air, N_2_ flow, and vacuum were conducted (Figures 3d, S27, and S28). The PXRD peaks shifted to higher 2θ values with increasing temperature in all three cases. **fcu-3-Co(III)** was observed under a vacuum at 300 °C (Figure 3d). This phase was also observed under ambient air and N_2_ flow but at different temperatures (Table S3).

The above results indicate that oxidation from Co(II) to Co(III) has occurred in the presence of O_2_ (air) and oxidants or upon heating. This is consistent with the chemistry of Co(II) complexes, where oxidation can be triggered by O_2_ or H_2_O_2_.^9,24,50^ We propose the following reaction mechanism: (1) 8Co(II) + 6(μ_4_–OH) + 2H_3_O^+^ + 2O_2_ → 8Co(III) + 6(μ_4_-O) + 6H_2_O; (2) 8Co(II) + 6(μ_4_–OH) + 2H_3_O^+^ + 4H_2_O_2_ → 8Co(III) + 6(μ_4_-O) + 10H_2_O. Transformation from Co(II) to Co(III) was also achieved by heating under vacuum and N_2_ flow. Additional experiments were performed to gain further insight.

**Figure 4:**
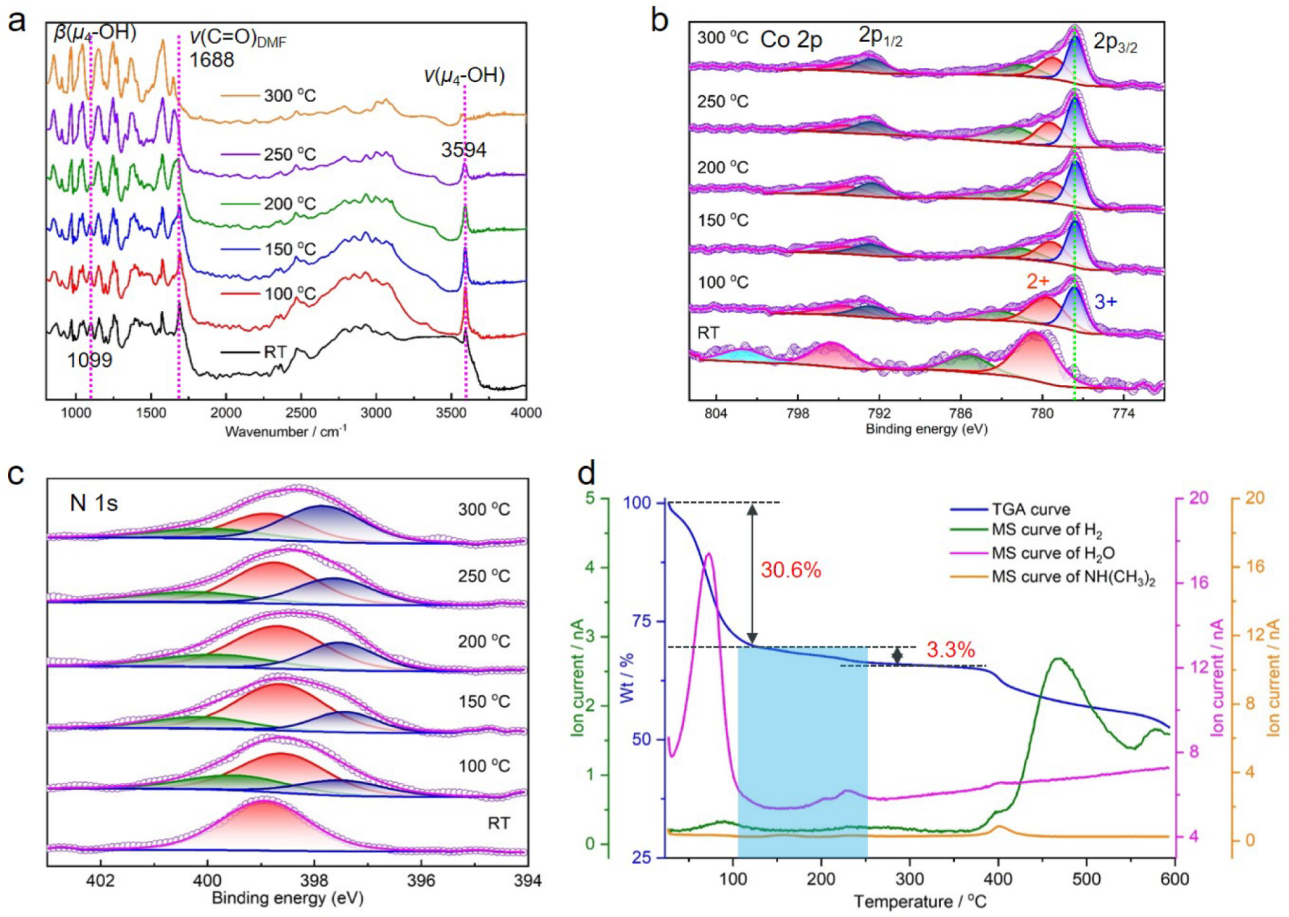
*In situ* spectroscopic study of Co(II) to Co(III) conversion in **fcu-3-Co**, experiments conducted on the MeOH exchanged and air dried **fcu-3-Co(II)** sample. (a) *In situ* IR spectra. *In situ* XPS: (b) Co 2*p* and (c) N 1*s* spectra. (d) TGA-MS curves.

That crystals of **fcu-3-Co(II)-β** and **fcu-3-Co(III)** after treatments retained crystallinity allowed us to study them by SCXRD, which revealed that **fcu-3-Co(II)-β** retained space group Fmm (Table S1). The framework structure of **fcu-3-Co(II)-β** contracted relative to that of **fcu-3-Co(II)**, with unit cell volume reduced from 16,262.2(2) to 15,560.6(9) Å attributable to shortening of Co–O and Co–N bond distances from 2.2076(28)/2.0620(23) Å to 2.1502(13)/1.9919(24) Å (Table S2 and S32). That partial oxidation of Co(II) to Co(III) has occurred is supported by *in situ* X-ray photoelectron spectroscopy (XPS) spectra (Figure 4b). The quality of **fcu-3-Co(III)** crystals had degraded after long-term air exposure, oxidants treatment, and heating, so only the unit cell parameters of **fcu-3-Co(III)** could be determined, giving a unit cell volume of 13,882.4(6) Å^3^. Nevertheless, SCXRD data were obtained after water vapor adsorption/desorption cycling measurements, which revealed a further unit cell volume reduction to 13,730.0(1) Å^3^, 15% less than that of **fcu-3-Co(II)-**α****. The Co–O and Co–N bond distances further decreased to 1.9309(49) and 1.8537(44) Å, along with protrusion of the μ_4_-O^2–^ moieties on the faces of the Co_8_ cube. In Co(II) compounds, typical Co–O/N bond lengths are expected to be around 2.1 Å, while bond lengths for Co(III) should be close to 1.9 Å, both in agreement with values of **fcu-3-Co**.^28,51,52^ Consequently, the volume of the Co_8_ cube contracted from 27.956 Å^3^ (**fcu-3-Co(II)**) to 16.856 Å^3^ (**fcu-3-Co(III)**), corresponding to a 40% shrinkage (Figure S32).

This redox-induced phase transformation was also studied by *in situ* infrared (IR) spectroscopy (Figure 4a). The diagnostic stretching bands of μ_4_–OH centered at 3594 cm^–1^ weakened at 200 °C and shifted to 3587 cm^–1^ at 250 °C.^53^ Furthermore, the bending band at 1099 cm^–1^ showed a similar trend, indicating a transformation upon heating. *In situ* XPS spectra were collected on **fcu-L-Co(II)** to determine the oxidation state of cobalt ions at different temperatures (Figures 4b–c and S33–S36). In the cobalt spectra of **fcu-3-Co(II)**, the peak at 777.6 eV that appeared at 100 °C was assigned to Co(III), which was not present in the room temperature (RT) sample (Figure 4b). Compared with the Co(II) signals of 780.4 and 779.4 eV as observed in the RT and heated samples, this peak is of lower energy and is consistent with reported values for Co(III).^19,24^ In addition to the peak at 531.2 eV, a peak of lower energy (528.8 eV) appeared in the oxygen spectra collected for heated samples (Figure S33b). In the nitrogen spectra, a new peak appeared at a lower energy of 397.6 eV as well (Figure 4c). These results support the proposed cobalt oxidation states conversion in the three forms of **fcu-3-Co**.

Thermogravimetric analysis–mass spectrometry (TGA-MS) analysis was also performed to probe the species released upon heating the **fcu-3-Co(II)** sample (Figures 4d and S37–S40). A signal for water was observed when **fcu-3-Co(II)** was heated at 63 °C, which was assigned to physisorbed water in pores. At 246 °C, both water and trace hydrogen were released (Figure 4d). Redox processes involving electron transfer within confined environments along with hydrogen evolution have been reported by Hashimoto’s and Hupp’s groups.^54−56^ Based on our experimental findings and literature reports, we propose a different transformation pathway under vacuum/inert conditions, in which water and hydrogen were generated as byproducts: 8Co(II) + 6(μ_4_–OH) + 2H_3_O^+^ → 8Co(III) + 6(μ_4_-O) + 2H_2_O + 4H_2_. When Co(II) converted to Co(III), one electron was transferred to the proton of μ_4_–OH or countercation H_3_O^+^ to yield hydrogen. Meanwhile, the μ_4_–OH moieties became μ_4_-O and H_3_O^+^ was converted to H_2_O, forming a neutral [Co(III)_8_(μ_4_-O)_6_(Pz)_12_] MBB in **fcu-3-Co(III)**. These signals were also observed in the **fcu-3-Co(II)** sample without MeOH exchange, however, the spectra are more complicated due to the thermal decomposition of residual DMF (Figure S39). By contrast, there was no hydrogen release monitored for **fcu-3-Co(III)**, with only a water signal observed at the same temperature (Figure S40).

We utilized superconducting quantum interference device (SQUID) magnetometry to quantify the content of Co(III) in **fcu-L-Co(III)** MOFs.^28,57,58^ Isothermal moment versus field (MvH) measurements were performed at 5 K, sweeping from −70 to 70 kOe and back, on both **fcu-L-Co(II)** and **fcu-L-Co(III)** samples (Figure S41). The results inferred low-spin 3*d*^7^ Co(II) ions (1.7–2.0 μ_B_, *S* = 1/2) in **fcu-L-Co(II)**, and predominantly high-spin 3d^6^ Co(III) (3.1–4.8 μ_B_, *S* = 2) in **fcu-L-Co(III)** with some residual Co(II) sites. Specifically, 63%, 79%, 98%, and 92% of Co(II) ions were calculated to convert to Co(III) in **fcu-1-Co(III)**, **fcu-2-Co(III)**, **fcu-3-Co(III)**, and **fcu-4-Co(III)**, respectively. **fcu-3-Co(III)** and **fcu-4-Co(III)** had therefore undergone almost complete oxidation from Co(II) to Co(III), while partial oxidation was observed for **fcu-1-Co(III)** and **fcu-2-Co(III)**. These results are consistent with the PXRD patterns of **fcu-1-Co(III)** and **fcu-2-Co(III)** that are characteristic of a Co(II)/Co(III) mixture (Figures S17–20). This behavior is different from that of Co_2_Cl_2_BTDD and Co_2_Cl_2_*X*_2_BTDD comprising Co-based RBBs.^28,57^ Coordinating with three nitrogen and three oxygen atoms in an octahedral geometry, the 3*d*^7^ electrons of MBB Co(II) adopt a low spin configuration: three pairs of electrons fill in three *t*_2g_ orbits, and one unpaired electron in an *e*_g_ orbit (Figure S32d).^59^ By contrast, Co(III) has one pair of electrons in a *t*_2g_ orbital, and four unpaired electrons, indicative of high-spin 3*d*^6^ (Figure S32d). To the best of our knowledge, this study details the first time a spontaneous and reversible cluster MBB Co(II) to Co(III) transformation has been monitored by SCXRD in a MOF and also reports a rare example of a MOF that breathes through MBB transformation.

### Water Vapor Sorption

**Figure 5:**
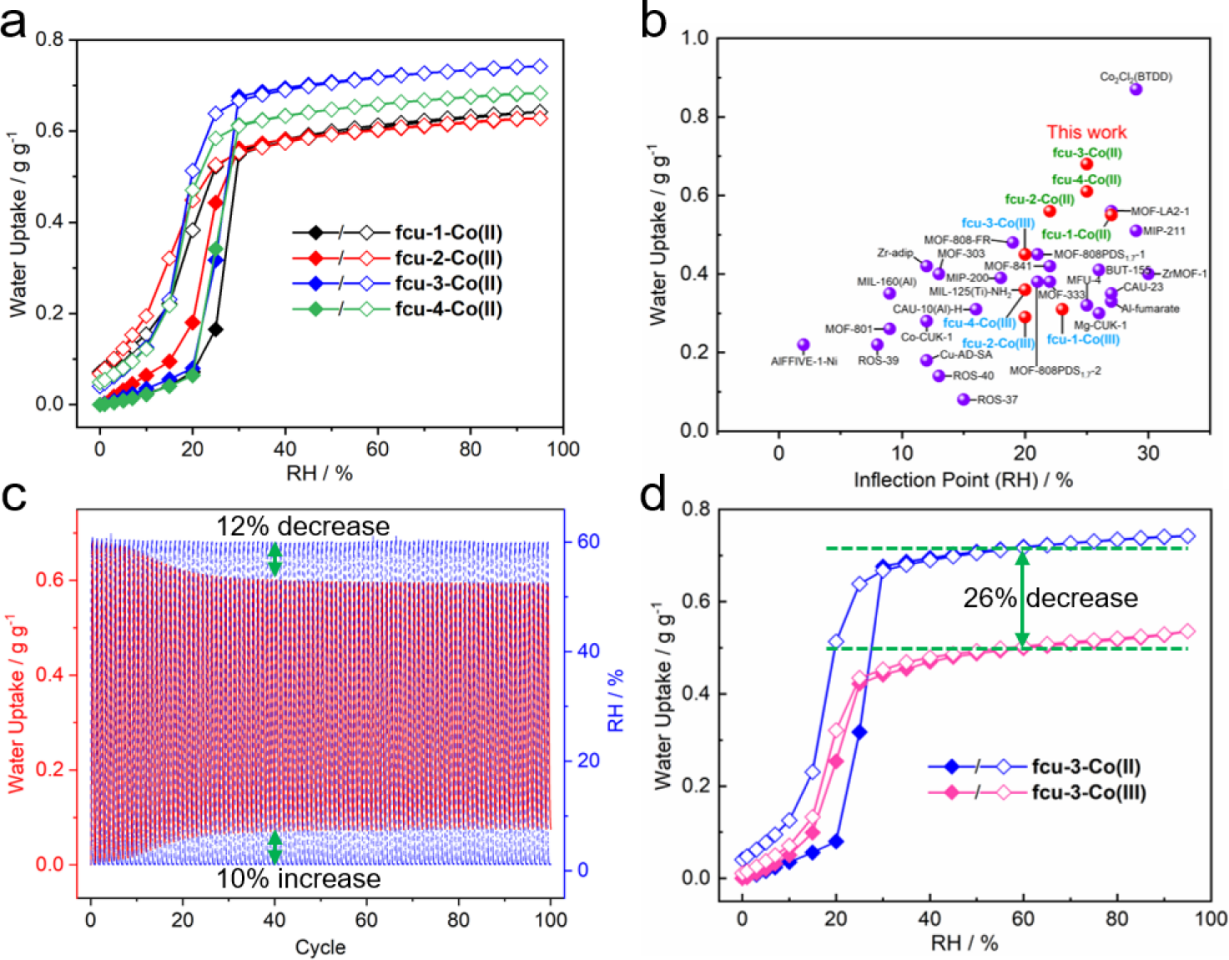
(a) Water vapor sorption isotherms of **fcu-L-Co(II)** at 298 K. (b) Comparison of water uptake under 30% RH at 298 K and the inflection point between **fcu-L-Co(II)** (green)**/fcu-L-Co(III)** (blue) and representative materials (black). (c) Water cycling test of **fcu-3-Co(II)** for 0%–60% RH humidity swing at 298 K. (d) Water isotherms collected before and after the cycling test of **fcu-3-Co** at 298 K.

The structure of **fcu-L-Co(II)** features large cages with hydrophilic windows, which motivated us to investigate its water vapor sorption properties. Isotherms were collected on activated samples at 298 K. All four variants exhibited S-shaped isotherms with steep uptake at <30% relative humidity (RH) (Figure 5a). The maximum water uptake for **fcu-1-Co(II)**, **fcu-2-Co(II)**, **fcu-3-Co(II)**, and **fcu-4-Co(II)** was determined to be 0.64 g g^–1^, 0.63 g g^–1^, 0.74 g g^–1^, and 0.68 g g^–1^ at 95% RH, respectively. Among these materials, **fcu-3-Co(II)** exhibited the highest uptake of 0.68 g g^–1^ at 30% RH, which is second to Co_2_Cl_2_(BTDD) (0.83 g g^–1^) as reported by Dincă and coworkers.^60^ The uptake difference between adsorption at 30% RH and desorption at 5% RH is also high, calculated to be 0.44 g g^–1^ for **fcu-1-Co(II)**, 0.44 g g^–1^ for **fcu-2-Co(II)**, 0.60 g g^–1^ for **fcu-3-Co(II)**, and 0.53 g g^–1^ for **fcu-4-Co(II)** (Table S4). SCXRD analysis on the water loaded structure of **fcu-3-Co(II)**-**β** revealed that the adsorbed water molecules are located around the Co_8_ MBBs and N-functionalized ligands, sustained by hydrogen bonds (Figure S42 and Table S5). There are 72 water molecules determined per formula, corresponding to an uptake of 0.67 g g^–1^ at 50% RH.

Water vapor adsorption/desorption kinetics (0–30% and 0–60% RH) and cycling experiments (0–60% RH) were performed at 298 K (Figures 5c and S43–S49). Relative to the first cycle of **fcu-1-Co(II)**, a 9% decrease of the working capacity (0.44 to 0.40 g g^–1^) was observed after 100 cycles (Figure S47). A 17% decrease from 0.45 to 0.37 g g^–1^ was seen for **fcu-2-Co(III)** (Figure S48). A decrease of 22% (0.68 to 0.53 g g^–1^) was recorded for **fcu-3-Co** after 40 cycles, with a 10% increase of the initial mass and a 12% decrease in maximum uptake, both of which remained almost unchanged in subsequent cycles (Figure 5c). **fcu-4-Co** behaved similarly to **fcu-3-Co**, with a capacity decrease of 23% after 40 cycles (0.64 to 0.48 g g^–1^) (Figure S49). Among these variants, the working capacity of **fcu-3-Co** after cycling (**fcu-3-Co(III)**, 0.52 g g^–1^) was the highest (Figures 5c, S47–S49 and Table S4). The isotherms of **fcu-L-Co(III)** were also collected (Figures 5d,S50 and S51). Uptake of **fcu-3-Co(III)** after cycling was 0.50 g of g^–1^ at 60% RH, corresponding to a 26% decrease relative to that measured before cycling. This value is consistent with the total capacity decrease by 22% in cycling. In addition, the isotherms evolved to be reversible without initial hysteresis (Figure 5d). It should be noted that the density of **fcu-3-Co(III)** (0.878 g cm^–3^) is greater than that of **fcu-3-Co(II)** (0.744 g cm^–3^). The volumetric water capacities between 60% and 5% RH for the two phases are therefore comparable (0.50 g cm^–3^ for **fcu-3-Co(II)** and 0.46 g cm^–3^ for **fcu-3-Co(III)**). Meanwhile, the inflection point shifted to lower RH (from 25% to 20%) and framework stability improved in **fcu-3-Co(III)**, which are both advantageous to water vapor sorption.

The quality of samples after cycling measurements (**fcu-L-Co(III)**) was first examined using PXRD. Crystallinity was retained while PXRD peaks shifted to higher 2θ relative to the **fcu-L-Co(II)** phases, indicating framework contraction. This aligns with the VT-PXRD data (Figures S8–S11 and 3). We also analyzed the single crystal structure of **fcu-3-Co(III)**. After cycling, the unit cell volume of **fcu-3-Co(III)** decreased by 15% (Table S1). Such contraction accounts for the maximum water uptake decrease of 12% in the cycling experiments (Figure 5c). These results indicate that ambient adsorption/desorption cycles lead to controlled conversion of Co(II) to Co(III).

With respect to the initial mass increase during water vapor sorption cycling, as revealed by SCXRD, water molecules reside around the Co_8_ MBB of **fcu-3-Co(III)** and account for ∼10% of the mass of the framework. This value is consistent with the mass increase being the result of a pocket that strongly binds water molecules. **fcu-3-Co(III)** after cycling was reactivated at 100 °C and a second round of cycling was conducted to test 0–60% RH pressure swing at RT for 40 cycles. Thereafter, *in situ* activations at 100, 150, and 200 °C were performed successively, each followed by two cycles of pressure swing at RT (Figures S52 and S53). The results of these tests indicate that this phase had a water uptake of 0.58 g g^–1^ in the first cycle, decreasing to 0.56 g g^–1^ after 40 cycles along with an initial mass increase of 0.04 g g^–1^ (Figure S52). After activating the sample *in situ* at 150 °C, the initial mass came back to zero, while the uptake in the following adsorption was 0.55 g g^–1^ with a residual 0.02 g g^–1^ uptake after desorption, giving a working capacity of 0.53 g g^–1^ (Figures S52 and 53). Both capacity values agree with the final capacity (0.52 g g^–1^) in the first round of the cycling test. Overall, these results indicate that, whereas the phase after the first round of cycling exhibits almost constant water vapor sorption capacity, strongly adsorbed water molecules, which form multiple ···H··X (X = O or N) interactions with the surrounding ligands and MBBs (Figure S54) hinder complete desorption and require more energy for regeneration (Figures S52 and 53).

## Conclusion

In summary, a family of four pyrazolate **fcu** topology MOFs have been constructed with a 12-connected Co_8_ MBB through a crystal engineering approach. The octahedrally coordinated Co(II) cations are redox active and can spontaneously transform in air to Co(III) in a single crystal to single crystal fashion. Further, this oxidation reaction can be initiated by heat or conventional oxidants. The effects of oxidation upon water vapor sorption properties were as follows: gravimetric uptake was reduced; volumetric uptake was similar; the inflection point shifted to lower RH (from 25% to 20%); cycling stability improved. Co(II) to Co(III) conversion therefore enhanced the water vapor harvesting performance. To our knowledge, this work represents the first demonstration of spontaneous cobalt cluster MBB redox chemistry in a MOF and, thanks to retention of crystallinity and *in situ* spectroscopy, insight into the reversible phase transformations was gained. These findings are instructive for designing and discovering reusable solid-state redox materials for efficient gas and vapor capture and catalysis.

## Supporting Information Available

Materials and instruments, details of ligands’ and MOFs’ synthesis, general characterizations (FT-IR, TGA, PXRD, etc.), and additional tables and figures; X-ray data for **fcu-1-Co(II)-α**; X-ray data for **fcu-2-Co(II)-α**; X-ray data for **fcu-3-Co(II)-α**; X-ray data for **fcu-4-Co(II)-α**; X-ray data for **fcu-3-Co(II)-β**; X-ray data for **fcu-3-Co(III)-γ**; X-ray data for **fcu-3-Co(II)-β**-H_2_O (PDF)
