# Supplementary material for: Reversible Co(II)–Co(III) Transformation in a Family of Metal–Dipyrazolate Frameworks
Source: J Am Chem Soc. 2024 Oct 8;146(41):28320–8. doi: 10.1021/jacs.4c09173 (PMC11487582; doi:10.1021/jacs.4c09173)
Supplement: Supplementary file 1 — ja4c09173_si_001.pdf [file ja4c09173_si_001.pdf]

Reversible Co(II)-Co(III) Transformation in a Family of Metal-Dipyrazolate Frameworks

Xiang-Jing Kong,[1,2] Tao He,[*1,2] Andrey A. Bezrukov,[2] Shaza Darwish,[2] Guang-Rui Si,[1] Yong-Zheng Zhang,[3] Wei Wu,[1] Yingjie Wang,[1] Xia Li,[2] Naveen Kumar,[2] Jian-Rong Li,[*1] Michael J. Zaworotko[*2]

[1] Beijing Key Laboratory for Green Catalysis and Separation and Department of Chemical Engineering, College of Materials Science and Engineering, Beijing University of Technology, Beijing 100124, P. R. China

[2] Bernal Institute and Department of Chemical Sciences, University of Limerick, Limerick, V94 T9PX, Ireland.

[3] Shandong Provincial Key Laboratory of Monocrystalline Silicon Semiconductor Materials and Technology, College of Chemistry and Chemical Engineering, Dezhou University, Shandong, 253023, China.

Email: xtal@ul.ie; jrli@bjut.edu.cn; hetao@bjut.edu.cn

Contents

Section 1. Materials and Methods..S3

Section 2. Ligands Synthesis...S5

Section 3. MOFs Synthesis and Activation S9

Section 4. X-ray Crystallographic Analysis S11

Section 5. *In situ* Characterization S13

Section 6. Magnetic Properties...S14

Section 7. Water Vapor Adsorption S15

Section 8. Kinetics and Recyclability Tests S16

Section 9. Additional Figures and Tables S17

Section 10. References...S45

Section 1. Materials and Methods

Starting materials, reagents and solvents were purchased from commercial sources and used without further purification.

Powder X-ray Diffraction (PXRD). Diffractograms were recorded using a PANalytical Empyrean™ diffractometer equipped with a PIXcel3D detector operating in scanning line detector mode with an active length of 4 utilizing 255 channels. The diffractometer is outfitted with an Empyrean Cu LFF (long fine-focus) HR (9430 033 7310x) tube operated at 40 kV and 40 mA and CuKα radiation (λα = 1.540598 Å) was used for diffraction experiments. Continuous scanning mode with the goniometer in the theta-theta orientation was used to collect the data. Incident beam optics included the Fixed Divergences slit with anti-scatter slit PreFIX module, with a 1/8° divergence slit and a 1/4° anti-scatter slit, as well as a 10 mm fixed incident beam mask and a Soller slit (0.04 rad). Divergent beam optics included a P7.5 anti-scatter slit, a Soller slit (0.04 rad) and a Ni-β filter. In a typical experiment, 25 mg of sample was dried, ground into a fine powder and was loaded on a zero background silicon disks. The data was collected from 3°–40° (2θ) with a step-size of 0.0131303° and a scan time of 30 seconds per step. Crude data were analyzed using the X'Pert HighScore Plus™ software V 4.1 (PANalytical, The Netherlands).

Variable Temperature Powder X-ray Diffraction (VT-PXRD). Diffractograms at different temperature were recorded using a PANalytical X'Pert Pro-MPD diffractometer equipped with a PIXcel3D detector operating in scanning line detector mode with an active length of 4 utilizing 255 channels. Anton Paar TTK 450 stage coupled with the Anton Paar TCU 110 Temperature Control Unit was used to record the variable temperature diffractograms. The diffractometer is outfitted with an Empyrean Cu LFF (long fine-focus) HR (9430 033 7300x) tube operated at 40 S5 kV and 40 mA and CuKα radiation (λα = 1.54056 Å) was used for diffraction experiments. Continuous scanning mode with the goniometer in the theta-theta orientation was used to collect the data. Incident beam optics included the Fixed Divergences slit, with a 1/2° divergence slit and a Soller slit (0.04 rad). Divergent beam optics included a P7.5 anti-

scatter slit, a Soller slit (0.04 rad), and a Ni-β filter. In a typical experiment, 20 mg of sample was dried and loaded on a zero background sample holder made for Anton Paar TTK 450 chamber. The data were collected from 4°–40° (2θ) with a step-size of 0.0167113° and a scan time of 50 seconds per step. Crude data were analyzed using the X'Pert HighScore Plus™ software V 4.1 (PANalytical, The Netherlands).

Thermogravimetric Analysis (TGA). Thermograms were recorded under nitrogen using TGA instrument TA Q50 V20.13 Build 39. Aluminum pans and a flow rate of 60 $cm^3\ min^{-1}$ for the nitrogen gas were used for the experiments. The data was collected in the High Resolution Dynamic mode with a sensitivity of 1.0, a resolution of 4.0 and a temperature ramp of 10 $K\ min^{-1}$ up to 580 °C. As control experiments, a lower temperature ramp of 2 $K\ min^{-1}$ was used to collect data from room temperature to 500 °C under air and nitrogen flow, respectively. The air experiment was performed after heating the system at 500 °C for one hour and cooling to clean the sample chamber. The data was evaluated using the T.A. Universal Analysis suite for Windows XP/Vista Version 4.5A.

Gas Sorption Measurements. For gas sorption experiments, ultrahigh-purity gases were used as received from BOC Gases Ireland: research-grade He (99.999%), CO_2 (99.995%), and N_2 (99.998%). Adsorption experiments (up to 1 bar) for 77 K N_2 were performed on Micromeritics 3Flex surface area and pore size analyser 3500. Before sorption measurements, activation of all five MOFs was achieved by degassing the air-dried samples filtered from methanol on a SmartVacPrep™ using dynamic vacuum and heating for 6 h (each sample heated from RT to 373 K with a ramp rate of 10 °C). Brunauer-Emmett-Teller (BET) surface areas were determined from the N_2 adsorption isotherms at 77 K respectively, using the Micromeritics Microactive software. About 50 mg of activated samples were used for the measurements. The low temperature of 77 K was controlled by a Dewar filled with liquid N_2.

Section 2. Ligands Synthesis

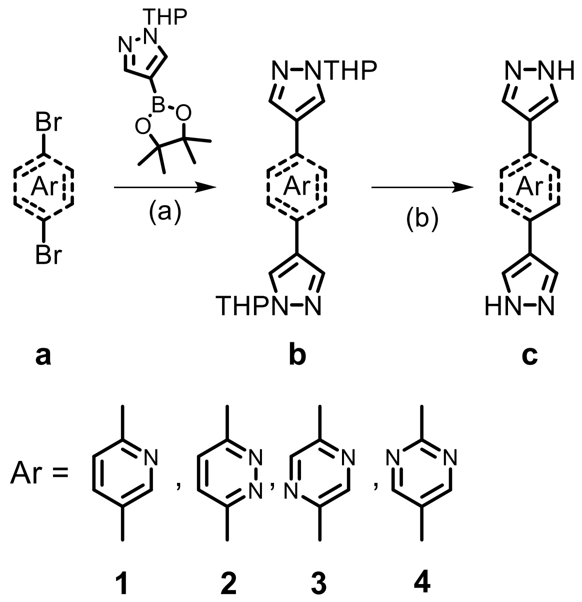

Scheme S1. General synthesis of dipyrazole ligands: (a) K_2CO_3, 1,4-dioxane, H_2O, $Pd(PPh_3)_4$; (b) EtOH, HCl.

General Suzuki coupling procedure.[S1] A mixture of **a** (10 mmol), 1-(tetrahydro-2H-pyran-2-yl)-4-(4,4,5,5-tetramethyl-1,3,2-dioxaborolan-2-yl)-1H-pyrazole (40 mmol), 1,4-dioxane (240 mL), water (60 mL), K_2CO_3 (40 mmol) and $Pd(PPh_3)_4$ (2 mmol) was stirred at 110 °C for 48 h under nitrogen atmosphere. After the starting materials were consumed, the solvent was removed and the residue was dissolved in ethyl acetate (300 mL), washed with water (300 mL × 2) and brine (300 mL), dried over anhydrous Na_2SO_4, filtered and concentrated. The crude product was purified by column chromatography to give **b**.

General deprotection procedure.[S1] The solution of **b** (5 mmol), ethanol (200 mL) and concentrated HCl (5 mL) was stirred at 50 °C for 12 h. The organic solvent was removed by rotary evaporation. The solid was dissolved in water (100 mL), and the pH was adjusted to around 10 by progressively adding saturated solution of Na_2CO_3. The mixture was filtered and washed with water (100 mL × 3). The solid was dried under vacuum at 60 °C to give **c**.

2.1 Synthesis of 2,5-di(1H-pyrazol-4-yl)pyridine (1)

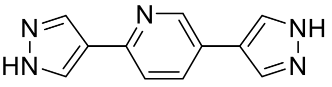

1: ^{1}H NMR (100 MHz, DMSO-d_6) δ 13.06 (2H, s), 8.80 (1H, d, J = 1.8 Hz), 8.29 (2H, s), 8.06 (2H, s), 7.97 (1H, dd, J = 8.2, 2.2 Hz), 7.68 (1H, d, J = 8.2 Hz).

2.2 Synthesis of 3,6-di(1*H*-pyrazol-4-yl)pyridazine (2)

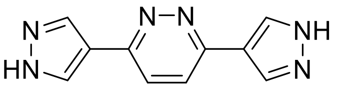

2: ^{1}H NMR (100 MHz, DMSO-d_6) δ 13.24 (2H, s), 8.48 (2H, s), 8.19 (2H, s), 7.96 (4H, s).

2.3 Synthesis of 2,5-di(1*H*-pyrazol-4-yl)pyrazine (3)

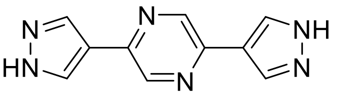

3: ^{1}H NMR (100 MHz, DMSO-d_6) δ 8.78 (2H, s), 8.03 (4H, s), 5.08 (2H, s).

2.4 Synthesis of 2,5-di(1H-pyrazol-4-yl)pyrimidine (4)

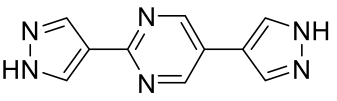

4: ^{1}H NMR (100 MHz, DMSO-d_6) δ 13.17 (2H, s), 9.01 (2H, s), 8.23 (4H, s).

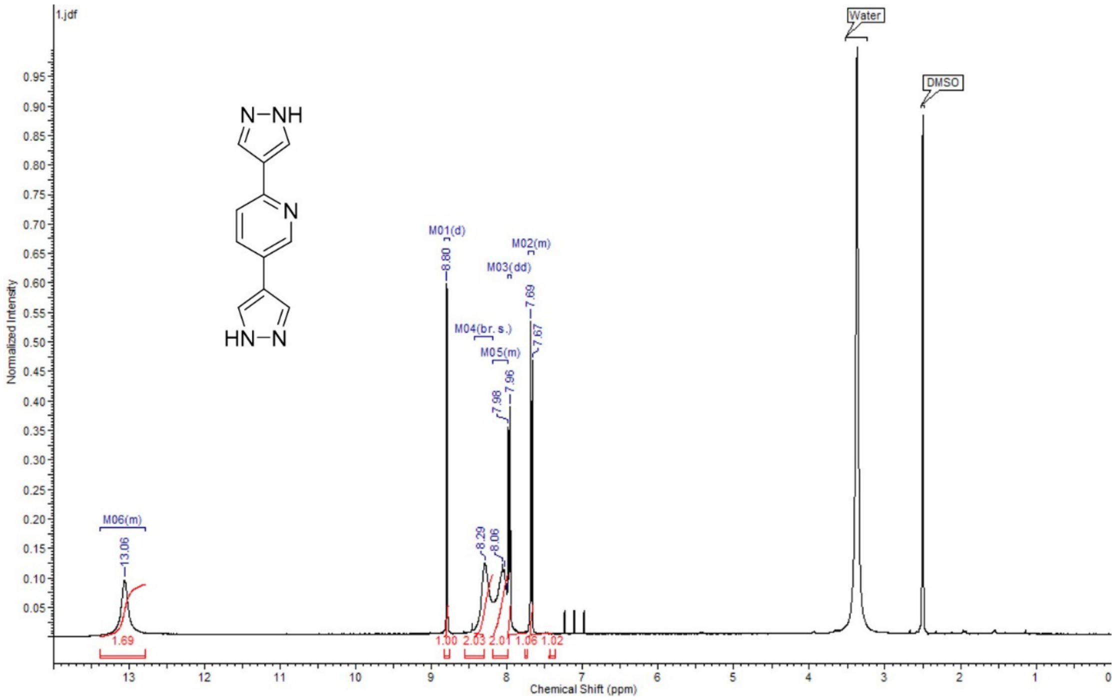

Figure S1 ^{1}H NMR of **1**.

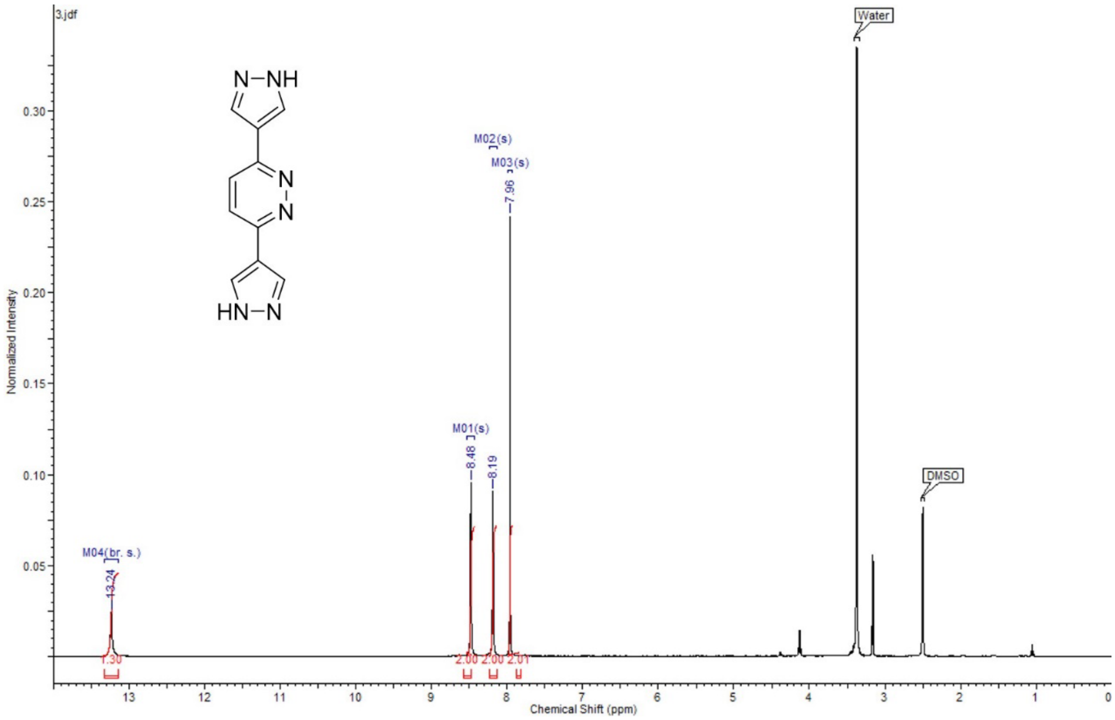

Figure S2 ^{1}H NMR of **2**.

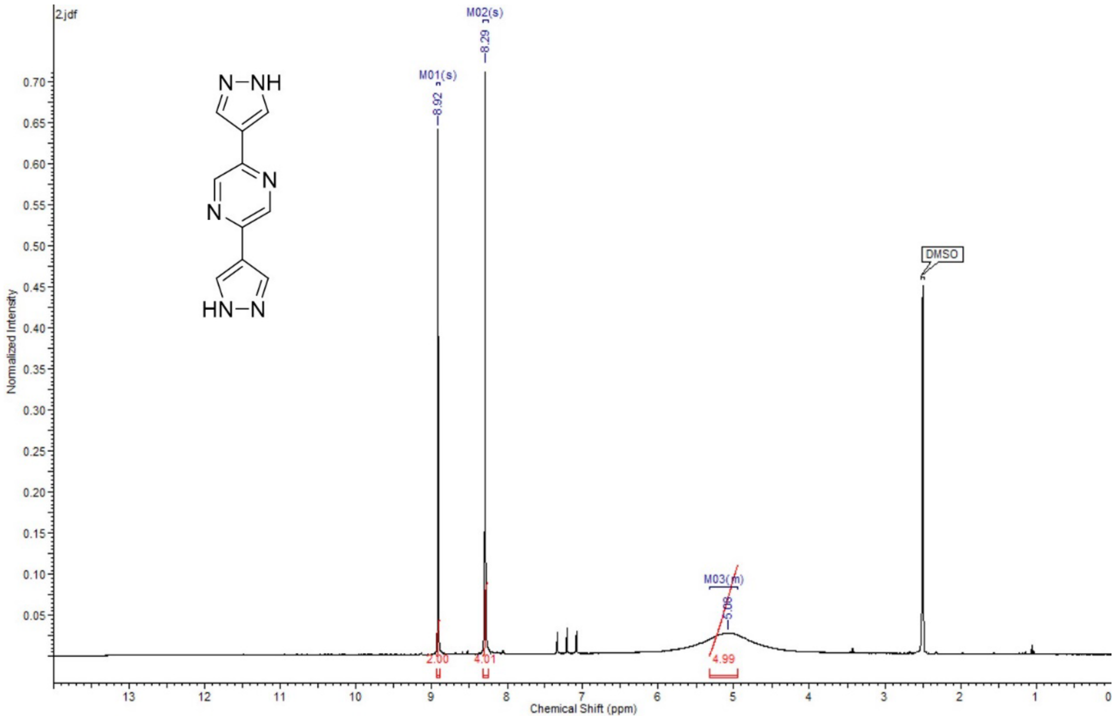

Figure S3 ^{1}H NMR of **3**.

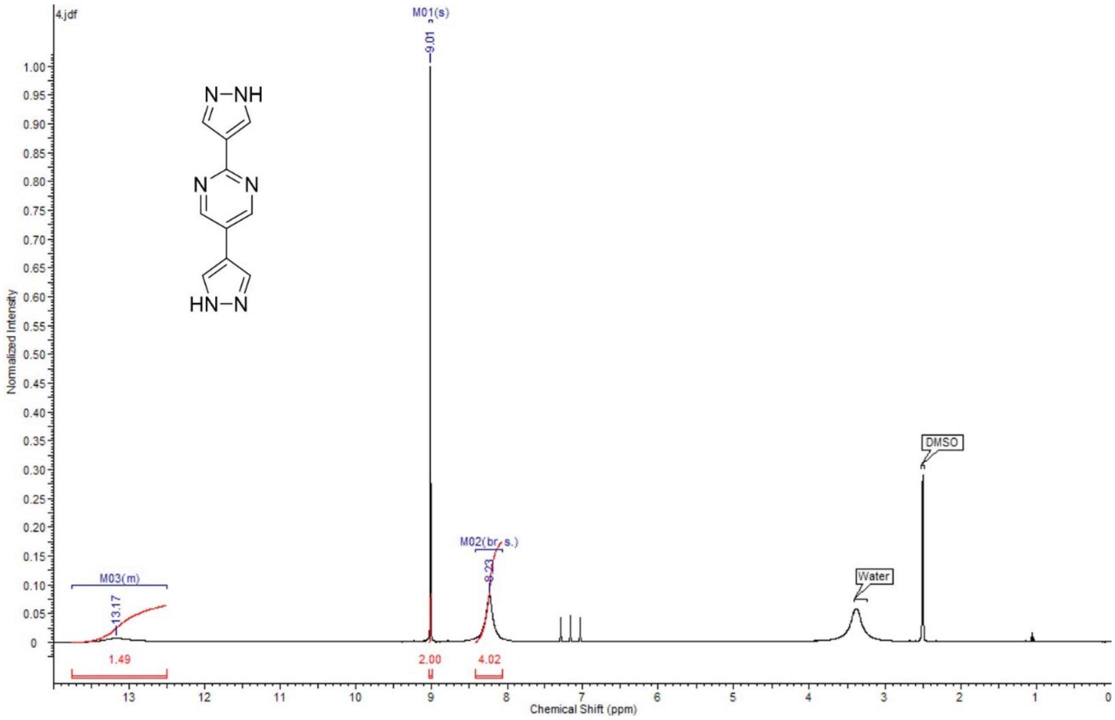

Figure S4 ^{1}H NMR of **4**.

Section 3. MOFs Synthesis and Activation

Based on the dipyrazolole ligands **1-4**, four MOFs, including **fcu-1-Co(II)** to **fcu-4-Co(II)**, have synthesized under solvothermal conditions, respectively. ***Notes***: *Using ligands from different batches may require adjustment to obtain pure phases, with the corresponding counterions being transformed into dimethylammonium cations.*

Synthesis of **fcu-1-Co(II)** (BUT-40).

Ligand **1** (0.14 mmol, 30 mg) and cobalt acetylacetonate (0.20 mmol, 50 mg) were dissolved under ultrasound in 10 mL DMF in a 20-mL glass vial. Next, 5.0 mL of deionized water was added to the mixture. The vial was then tightly sealed and the mixture sonicated for another 15 min. The resulting suspension was heated in a traditional oven at 120 °C for 3 days. After cooling naturally to room temperature, orange crystals were filtered off and washed with DMF (2 × 15 mL) and methanol (9 × 15 mL) with short cycles of ultrasonic treatment to remove amorphous solids. The crystals (10 mg of activated sample, 35% yield based on the **1** ligand) were collected by filtration and dried in air.

Synthesis of **fcu-2-Co(II)** (BUT-50).

Ligand **2** (0.14 mmol, 30 mg) and cobalt acetylacetonate (0.20 mmol, 50 mg) were dissolved under ultrasound in 10 mL DMF in a 20-mL glass vial. Next, 4.0 mL of deionized water was added to the mixture. The vial was then tightly sealed and the mixture sonicated for another 15 min. The resulting suspension was heated in a traditional oven at 120 °C for 3 days. After cooling naturally to room temperature, orange crystals were filtered off and washed with DMF (2 × 15 mL) and methanol (9 × 15 mL) with short cycles of ultrasonic treatment to remove amorphous solids. The crystals (13 mg of activated sample, 42% yield based on the **2** ligand) were collected by filtration and dried in air.

Synthesis of **fcu-3-Co(II)** (BUT-60).

Ligand **3** (0.14 mmol, 30 mg) and cobalt acetylacetonate (0.20 mmol, 50 mg) were

dissolved under ultrasound in 10 mL DMF in a 20-mL glass vial. Next, 3.0 mL of deionized water was added to the mixture. The vial was then tightly sealed and the mixture sonicated for another 15 min. The resulting suspension was heated in a traditional oven at 120 °C for 3 days. After cooling naturally to room temperature, orange crystals were filtered off and washed with DMF (2 × 15 mL) and methanol (9 × 15 mL) with short cycles of ultrasonic treatment to remove amorphous solids. The crystals (15 mg of activated sample, 49% yield based on the **3** ligand) were collected by filtration and dried in air.

Synthesis of **fcu-4-Co(II)** (BUT-68).

Ligand **4** (0.14 mmol, 30 mg) and cobalt acetylacetonate (0.20 mmol, 50 mg) were dissolved under ultrasound in 10 mL DMF in a 20-mL glass vial. Next, 5.0 mL of deionized water was added to the mixture. The vial was then tightly sealed and the mixture sonicated for another 15 min. The resulting suspension was heated in a traditional oven at 120 °C for 3 days. After cooling naturally to room temperature, orange crystals were filtered off and washed with DMF (2 × 15 mL) and methanol (9 × 15 mL) with short cycles of ultrasonic treatment to remove amorphous solids. The crystals (12 mg of activated sample, 39% yield based on the **4** ligand) were collected by filtration and dried in air.

Sample Activation.

Prior to gas adsorption tests, all the samples (about 50 mg for each) were immersed into 15 mL of DMF at 60 °C for 24 h, when fresh DMF was exchanged after 12 h. These samples were then harvested carefully by decanting and next soaked into 15 mL of methanol at room temperature for another 72 h, during which time fresh methanol was exchanged thrice every day. When solvent exchange finished, the samples were loaded in a sample tube and degassed under high vacuum at an optimal heated temperature of 80 °C for 6 h.

Section 4. X-ray Crystallographic Analysis

Single crystal X-ray diffraction data of all the crystals were collected on a Bruker Quest diffractometer equipped with a IμS microfocus X-ray source (Cu Kα, λ = 1.54178 Å; Mo Kα, (λ = 0.71073 Å) and CMOS detector. APEX4 was used for collecting, indexing, integrating and scaling the data.[S2] Open-flow nitrogen attachment with Oxford Cryosystem was used for low temperature measurements. Absorption correction was performed by multi-scan method.[S3] Space groups were determined using XPREP as implemented in APEX4.[S4] The datasets were corrected by empirical absorption correction using spherical harmonics, implemented in the SCALE3 ABSPACK scaling algorithm.[S5] All the structures were solved using direct methods and refined by full-matrix least-squares on F^2 with anisotropic displacement using the SHELXTL software package.[S6] Non-hydrogen atoms on frameworks were refined with anisotropic displacement parameters during the final cycles. The hydrogen atoms on frameworks were positioned geometrically or added according to theoretical models, and assigned isotropic displacement parameters to ride on their respective parent atoms. Some N, C, and O atoms in the structure of MOFs were disordered and treated by occupancy refinements. The volume fractions of disordered solvents in pores could not be modeled in terms of atomic sites, but were treated by using the MASK routine in the Olex2 software package.[S7-8] Crystallographic data for all the MOFs reported in this paper, including **fcu-1-Co(II)-α** to **fcu-4-Co(II)-α**, **fcu-3-Co(II)-β**, **fcu-3-Co(III)-γ**, and **fcu-3-Co(II)-β-H_2O**, are summarized in Table S1 and S2. Crystal structures are deposited to the Cambridge Crystallographic Data Centre (CCDC 2323663-2323668, 2384392).

Table S1 Crystallographic data and structure refinements for **fcu-L-Co**.

Name	**fcu-1-Co(II)-α**	**fcu-2-Co(II)-α**	**fcu-3-Co(II)-α**	**fcu-4-Co(II)-α**	**fcu-3-Co(II)-β**	**fcu-3-Co(III)-γ**
Formula	$C_{66}H_{62}Co_8N_{30}O_{12}$	$C_{60}H_{56}Co_8N_{36}O_{12}$	$C_{60}H_{56}Co_8N_{36}O_{12}$	$C_{72}H_{86}Co_8N_{42}O_6$	$C_{60}H_{54}Co_8N_{36}O_{12}$	$C_{60}H_{48}Co_8N_{36}O_{12}$
M.W.	1938.89	1944.81	1944.81	2107.23	1942.80	1936.75
Crystal system	Cubic	Cubic	Cubic	Cubic	Cubic	Cubic
Space group	$Fm\bar{3}m$	$Fm\bar{3}m$	$Fm\bar{3}m$	$Fm\bar{3}m$	$Fm\bar{3}m$	$Fm\bar{3}m$
a/ Å	25.4408(2)	25.3104(2)	25.3353(1)	25.3331(3)	24.9656(5)	23.9455(9)
b/ Å	25.4408(2)	25.3104(2)	25.3353(1)	25.3331(3)	24.9656(5)	23.9455(9)
c/ Å	25.4408(2)	25.3104(2)	25.3353(1)	25.3331(3)	24.9656(5)	23.9455(9)
α/ °	90	90	90	90	90	90
β/ °	90	90	90	90	90	90
γ/ °	90	90	90	90	90	90
V/ Å^3	16466.2(4)	16214.3(4)	16262.2(2)	16257.9(6)	15560.6(9)	13730.0(1)
Void/ %	61.2	61.8	61.1	53.4	62.6	61.0
D_C /g cm^{-3}	0.779	0.791	0.789	0.811	0.824	1.061
μ /mm^{-1}	6.474	6.574	6.555	6.545	6.851	7.873
T/ K	293	293	293	100	150	100
Reflections collected	3606	3387	7389	12358	12050	25113
Independent reflections	854<br>R_{int} = 0.0331	850<br>R_{int} = 0.0352	863<br>R_{int} = 0.0338	836<br>R_{int} = 0.0943	830<br>R_{int} = 0.0874	715<br>R_{int} = 0.3774
Goodness-of-fit on F^2	1.094	1.090	1.069	1.138	1.024	1.168
R_1[a], wR_2[b] [$I > 2\sigma(I)$]	R_1 = 0.0504<br>wR_2 = 0.1511	R_1 = 0.0625<br>wR_2 = 0.1883	R_1 = 0.0534<br>wR_2 = 0.1540	R_1 = 0.0612<br>wR_2 = 0.1727	R_1 = 0.0622<br>wR_2 = 0.1846	R_1 = 0.1714<br>wR_2 = 0.4385
R_1[a], wR_2[b] (all data)	R_1 = 0.0555<br>wR_2 = 0.1581	R_1 = 0.0694<br>wR_2 = 0.1975	R_1 = 0.0582<br>wR_2 = 0.1609	R_1 = 0.0680<br>wR_2 = 0.1824	R_1 = 0.0680<br>wR_2 = 0.1936	R_1 = 0.2298<br>wR_2 = 0.4976
Largest diff. peak and hole (e.Å^{-3})	0.62/–0.42	0.93 / –0.54	0.55 / –0.40	1.08/–0.65	0.83/–0.34	1.50/–1.75

[a] $R_1=\Sigma||F_o|-|F_c|| / \Sigma|F_o|$

[b] $wR_2=\{\Sigma[w(F_o^2-F_c^2)^2]/[w(F_o^2)^2]\}^{1/2}$, $[F_o>4\sigma(F_o)]$

Table S2 Coordination bond length and Co-Co distance for **fcu-L-Co**.

Name	**fcu-1-Co(II)-α**	**fcu-2-Co(II)-α**	**fcu-3-Co(II)-α**	**fcu-4-Co(II)-α**	**fcu-3-Co(II)-β**	**fcu-3-Co(III)-γ**
Co-O	2.2066(10)	2.2041(32)	2.2076(28)	2.1971(33)	2.1502(13)	1.9309(49)
Co-N	2.0619(23)	2.0644(29)	2.0620(23)	2.0561(28)	1.9919(24)	1.8537(44)
Co-Co	3.0346(7)	3.0297(7)	3.0352(7)	3.0192(7)	2.9454(7)	2.5636(14)
Pore diameter / Å	8.8 and 17.6	8.8 and 17.6	8.8 and 17.6	8.8 and 17.6	8.3 and 16.6	7.8 and 15.6

Section 5. *In situ* Characterization

In situ Infrared (IR) Spectroscopy. In situ IR measurements were performed on a Nicolet iS50 FTIR spectrometer using a liquid N_2-cooled DTGS detector. The sample (~5 mg) of air-dried **fcu-3-Co(II)** filtered from mother liquid was pressed to a KBr pellet, which was then placed into the testing cell. The tests were performed under nitrogen atmosphere in a temperature range of 25-300 ^{o}C (temperature ramp of 10 K / min) and a wavelength range of 400-4000 cm^{-1} (resolution of 2 nm and step size of 1 cm^{-1}).

In situ XPS. In situ XPS experiments were carried out with X-ray photoelectron spectroscopy (ThermoFisher K-Alpha/Thermo SCIENTIFIC ESCALAB 250Xi, ThermoFisher Scientific, USA) equipped with the X-ray lamp (Al Ka micrometer monochromator, hv = 1486.6 eV). The system was operating at 10^{-7} Pa. XPS ion depth profiling experiments were performed by Ar ion beam stripping technology using a differentially pumped ion gun (IQE-12/38 model) working at 100-4000 eV. All the reported binding energies (BE) data have been calibrated to the C1s peak at 284.6 eV. The sample (~5 mg) of air-dried **fcu-3-Co(II)** from mother liquid was tested under vacuum in a temperature range of 25-300 ^{o}C.

TGA-MS. TGA-MS measurements were carried out a TGA instrument SDT Q600 V20.9 Build 20 under nitrogen. Aluminum pans and a nitrogen flow rate of 100 cm^3 min^{-1} for the nitrogen gas were used for the experiments. The data was collected in the standard DSC-TGA mode with a sensitivity of 1.0, a resolution of 4.0 and a temperature ramp of 10 °C min^{-1} up to 600 °C. The data were evaluated using the T.A. Universal Analysis suite for Windows XP/Vista Version 4.5A. A Hiden HPR20 mass spectrometer was equipped to monitor the constituents of the outlet gas under the SGA mode with the electron-energy of 70 V. The mass value 44 was set for detecting $NH(CH_3)_2$, 18 for H_2O, and 2 for H_2, respectively, and the data were analyzed with MASsoft7. The sample (~10 mg) of air-dried **fcu-3-Co(II)** from mother liquid, air-dried **fcu-3-Co(II)** after MeOH exchange, and **fcu-3-Co(III)** were tested.

Section 6. Magnetic Properties

Magnetic susceptibility measurements were performed using a Quantum Design Magnetic Property Measurement System (MPMS®3) at 5 K, sweeping from −70 to 70 kOe and back.

The intrinsic magnetic moment could be calculated using the equation: $\mu = \sqrt{n(n+2)}$, where μ represents the intrinsic magnetic moment, and n denotes the number of unpaired electrons. For low-spin Co(II), the number of unpaired electrons, n, is 1, resulting in a calculated magnetic moment of 1.73 μ_B. For high-spin Co(III), the number of unpaired electrons, n, is 4, giving a calculated magnetic moment of 4.90 μ_B.

Note: In addition to the PXRD patterns of **fcu-L-Co(III)** samples after cycling water vapor sorption experiments, crystal data and water vapor sorption isotherms were also collected on **fcu-3-Co(III)** after cycling, and the latter were present in the text (Figure 5d). Given the cycling water vapor sorption experiments are time-consuming and are typically applicable on a small amount of sample (~5 mg per batch), we oxidized the **fcu-L-Co(II)** samples using chemical oxidant meta-chloroperoxybenzoic acid (*m*-CPBA, concentration: 5 mg/mL dichloromethane solution) in the revision to obtain enough **fcu-L-Co(III)** samples more quickly. The resulting **fcu-L-Co(III)** samples exhibited matched PXRD patterns with those after water vapor sorption cycling experiments, indicating retention of crystallinity and successful oxidation. This also suggests that water adsorption cycling can fully convert Co(II) to Co(III). The characterization performed on the oxidized **fcu-L-Co(III)** samples include PXRD, superconducting quantum interference device (SQUID) magnetometry, N_2 sorption isotherms at 77 K, and water vapor sorption isotherms.

Section 7. Water Vapor Adsorption

Water vapor sorption was performed using Adventure dynamic vapor sorption (DVS) instrument manufactured by Surface Measurement Systems. The instrument gravimetrically measures water vapor uptake using air as a carrier gas. Digital mass flow controllers regulate flows of dry and saturated gases. The saturated flow is created by passing dry air through a water bubbler. Relative humidity is generated by precisely mixing dry and saturated gas flows in desired, calibrated flow ratios which produce expected relative humidity. Pure water was used to generate water vapor for these measurements and temperature was maintained at 300 K or at 333 K by enclosing the system in a temperature-controlled incubator. The mass of the sample was determined by a high resolution microbalance Ultrabalance Low Mass with a precision of 0.01 μg. The microbalance has a symmetric configuration with both the sample pan and reference pan being exposed to the same gas and being kept at the same temperature, allowing negation of buoyancy and drag effects. The instrument is equipped with two such balances, allowing measurement of two samples in parallel. Prior to the measurement, each sample was activated in-situ in dry air at 100 °C for 60 minutes using the built-in preheater and consequently cooled to sorption temperature in 90 minutes. Isotherm measurements were performed on approximately 10 mg of sample powder. 400 sccm min^{-1} total flow was used for the measurements at 300 K and 50 sccm min^{-1} total flow was used for measurements at 333 K. We found the lower flow was necessary to achieve a stable relative humidity reading at higher temperatures, especially at higher humidity. The flow is split between two samples, thus the flow experienced by the sample was half of the total flow. For each isotherm point, $dm/dt < 0.05$ % min^{-1} for a minimum of 10 minutes was used as criteria of reaching equilibrium.

Section 8. Kinetics and Recyclability Tests

The kinetics and recycling tests were performed at 25 °C on Surface Measurement Systems DVS Intrinsic using air as a carrier gas to gravimetrically measure the uptake and loss of vapor (5 mg of sample for each experiment). The mass of the sample was determined by comparison to an empty reference pan and recorded by a high resolution microbalance with a precision of 0.1 μg. Kinetics was measured between two points: 0, 30%RH and 0, 60% RH successively with a convergence equilibrium criterion dm/dt = 0.01 %/min. The recycling tests were conducted for 100 cycles, each cycle consisting of 30 min adsorption step (60% RH) and 60 min desorption step (0% RH).

Section 9. Additional Figures and Tables

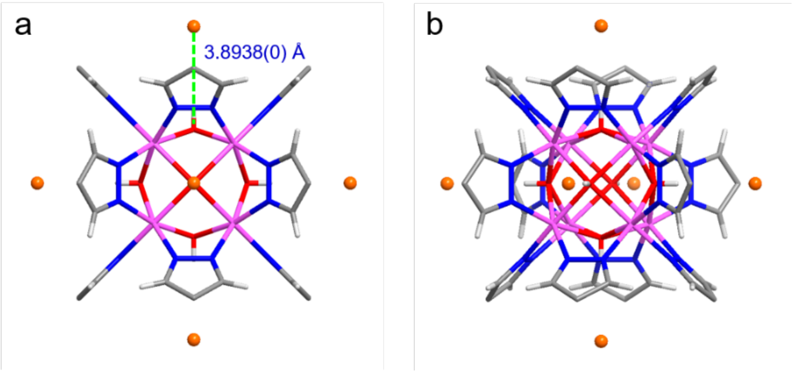

Figure S5 Structure of the Co_8 SBB with 6 countering H_3O^+/H_2O entities in **fcu-3-Co(II)**.

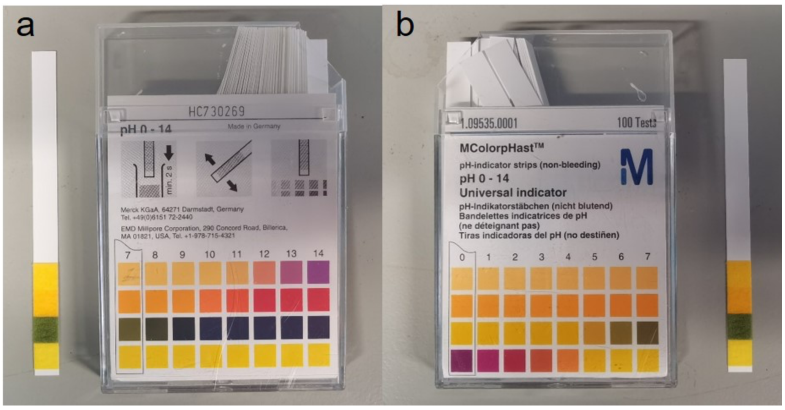

Figure S6 pH value measurements on the reaction mixture (a) before and (b) after solvothermal reactions.

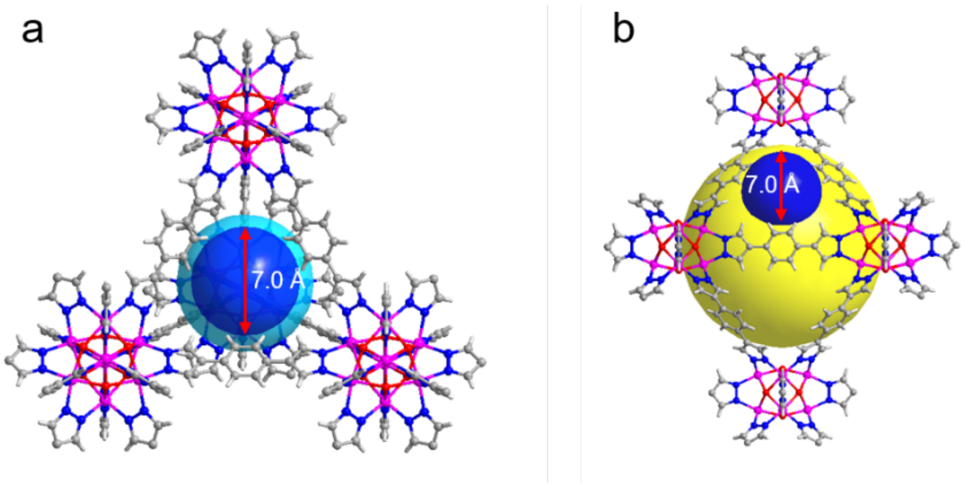

Figure S7 Structure of the (a) tetrahedron and (b) octahedron cages with hydrophilic windows in **fcu-3-Co(II)**.

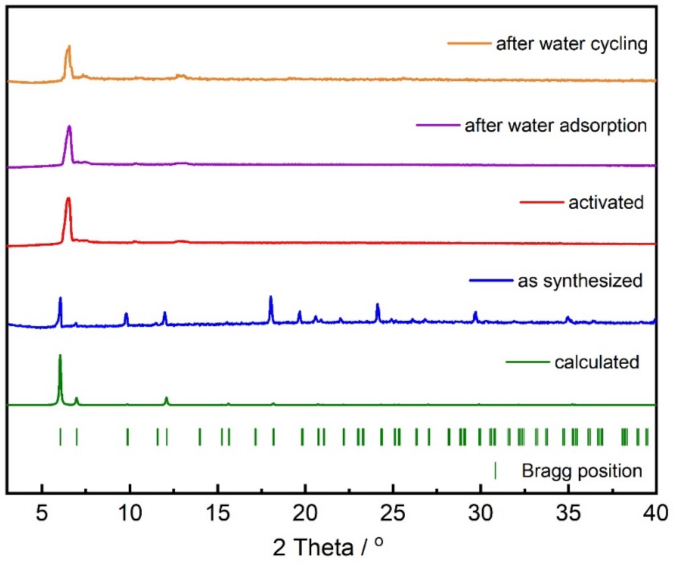

Figure S8 PXRD patterns of **fcu-1-Co**.

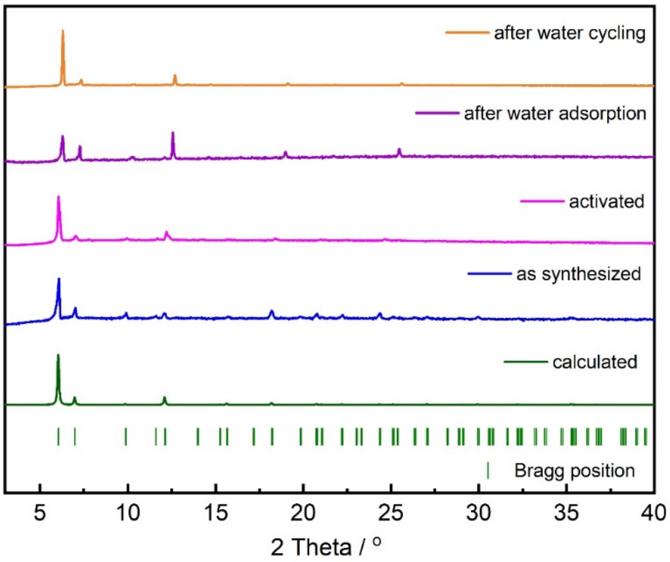

Figure S9 PXRD patterns of **fcu-2-Co**.

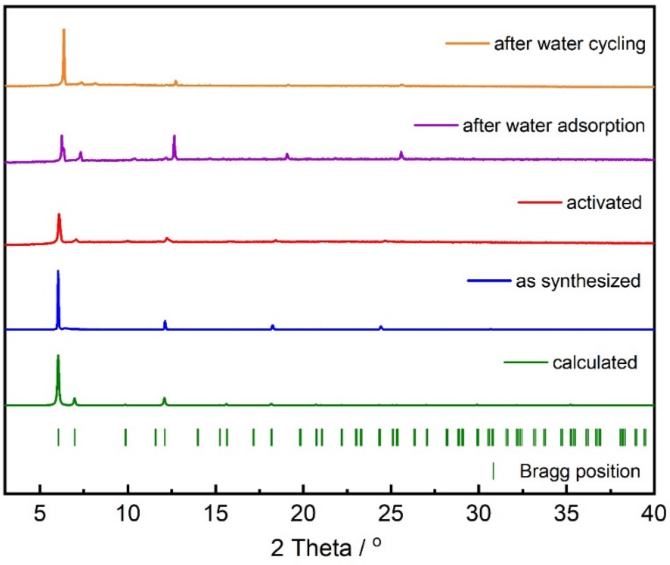

Figure S10 PXRD patterns of **fcu-3-Co**.

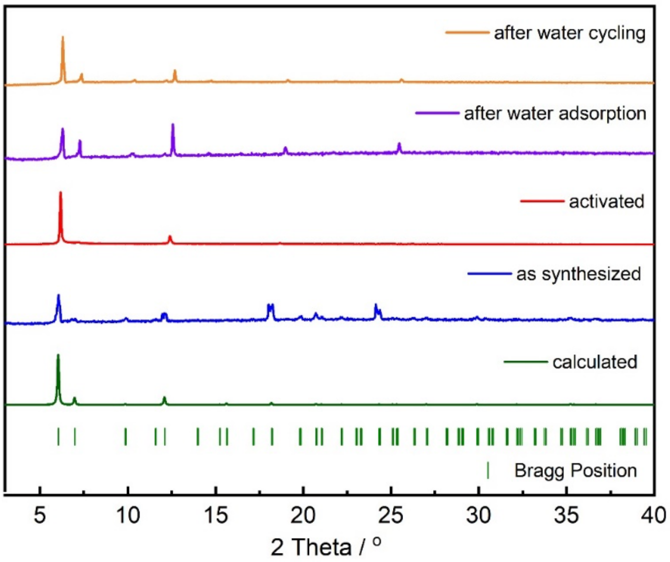

Figure S11 PXRD patterns of **fcu-4-Co**.

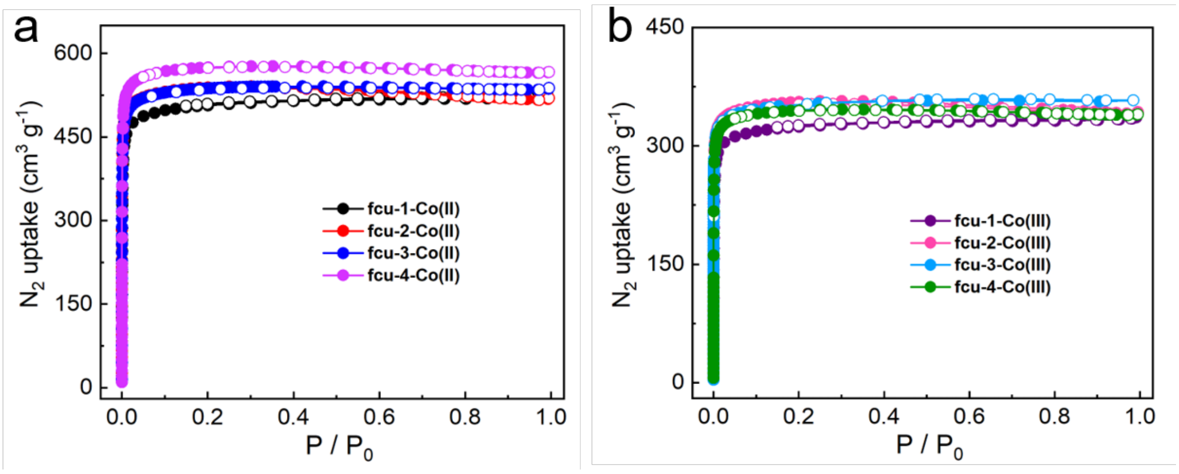

Figure S12 N_2 sorption isotherms of **fcu-L-Co(II)** and **fcu-L-Co(III)** at 77 K.

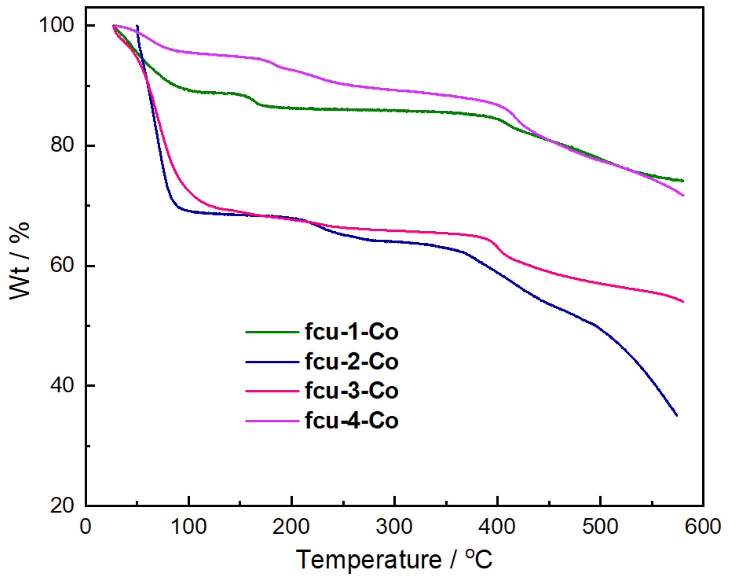

Figure S13 TGA curves of air-dried **fcu-L-Co(II)** after MeOH exchange.

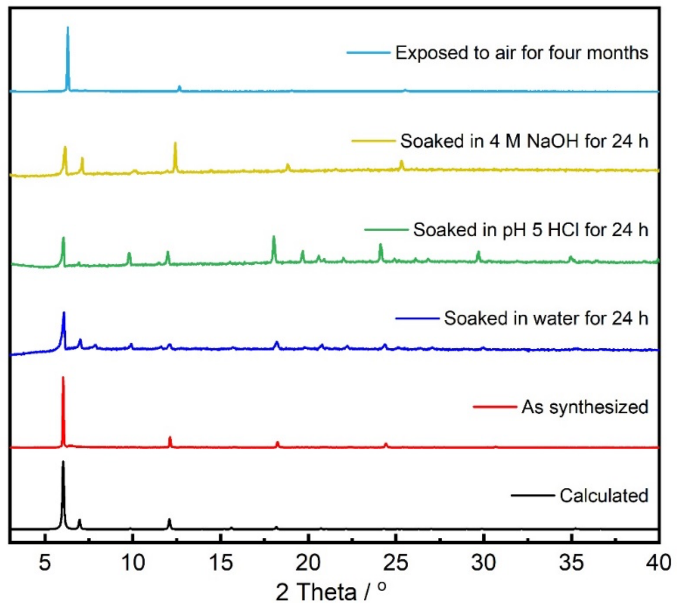

Figure S14 PXRD patterns of **fcu-3-Co** after different treatments.

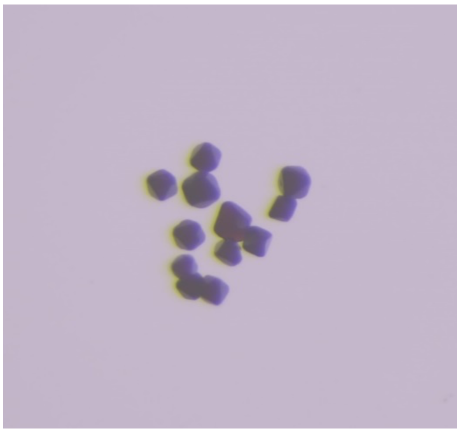

Figure S15 Image of **fcu-3-Co(II)-*β***.

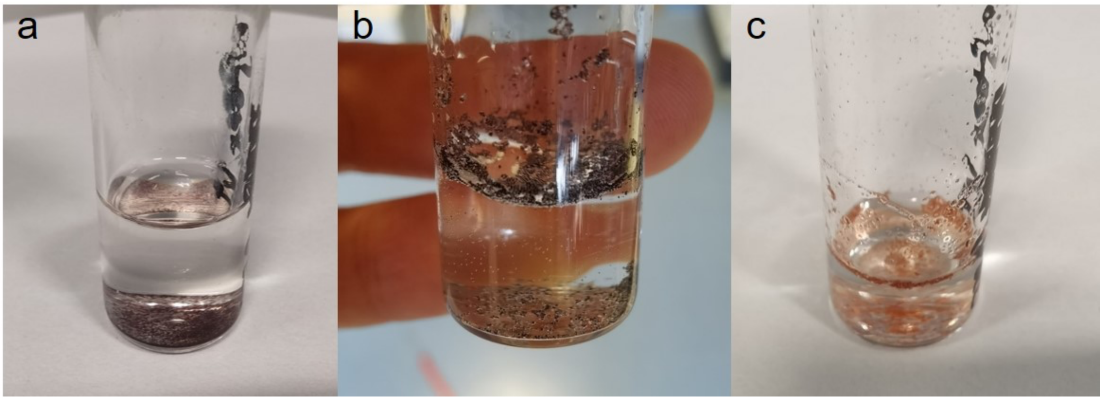

Figure S16 Crystals color change of **fcu-3-Co** from red of the MeOH exchange phase (**fcu-3-Co(II)-α**) to (a) dark purple after H_2O_2 oxidation (**fcu-3-Co(III)-γ**) with (b) bubbles released and then (c) back to red by reduction with $N_2H_4 \cdot H_2O$ (**fcu-3-Co(II)-α**).

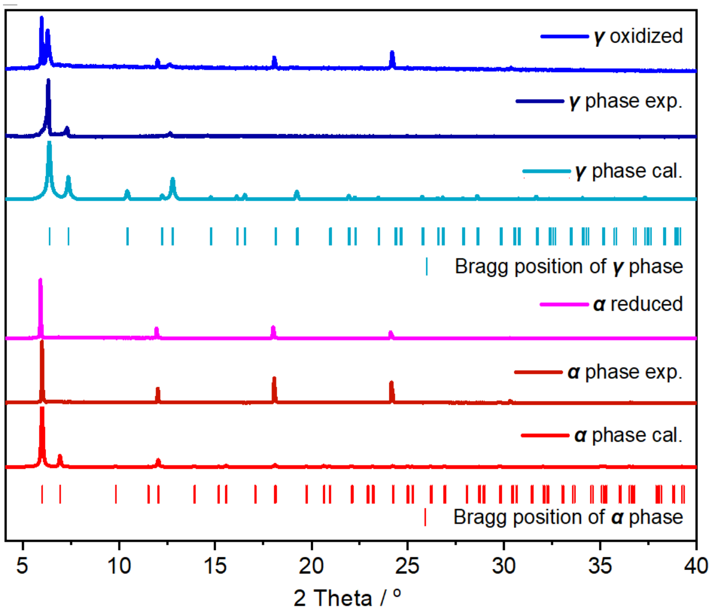

Figure S17 Comparison of PXRD patterns between calculated and experimental **fcu-1-Co(II),** as well as **fcu-1-Co(II)** obtained by reducing **fcu-1-Co(III)** with N_2H_4; comparison of PXRD patterns between the calculated **fcu-1-Co(III)**, **fcu-1-Co(III)** after cycling and **fcu-1-Co(III)** obtained by oxidizing **fcu-1-Co(III)** with *m*-CPBA.

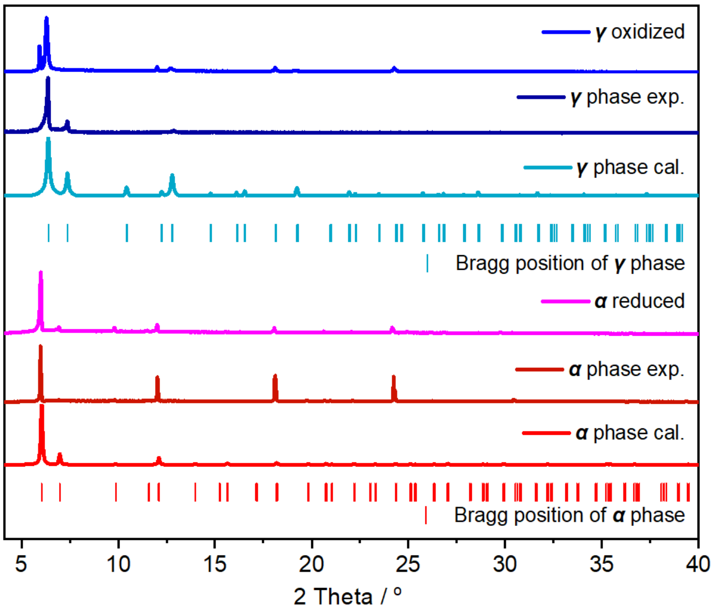

Figure S18 Comparison of PXRD patterns between calculated and experimental **fcu-2-Co(II),** as well as **fcu-2-Co(II)** obtained by reducing **fcu-2-Co(III)** with N_2H_4; comparison of PXRD patterns between the calculated **fcu-2-Co(III)**, **fcu-2-Co(III)** after cycling and **fcu-2-Co(III)** obtained by oxidizing **fcu-2-Co(III)** with *m*-CPBA.

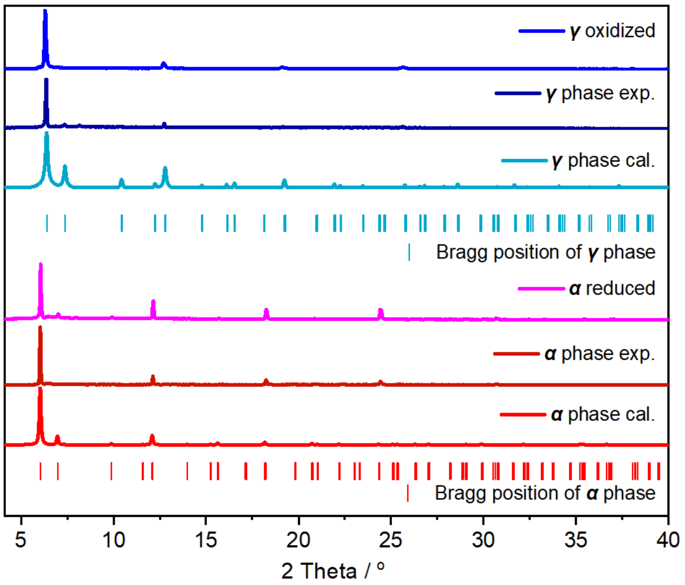

Figure S19 Comparison of PXRD patterns between calculated and experimental **fcu-3-Co(II),** as well as **fcu-3-Co(II)** obtained by reducing **fcu-3-Co(III)** with N_2H_4; comparison of PXRD patterns between the calculated **fcu-3-Co(III)**, **fcu-3-Co(III)** after cycling and **fcu-3-Co(III)** obtained by oxidizing **fcu-3-Co(III)** with *m*-CPBA.

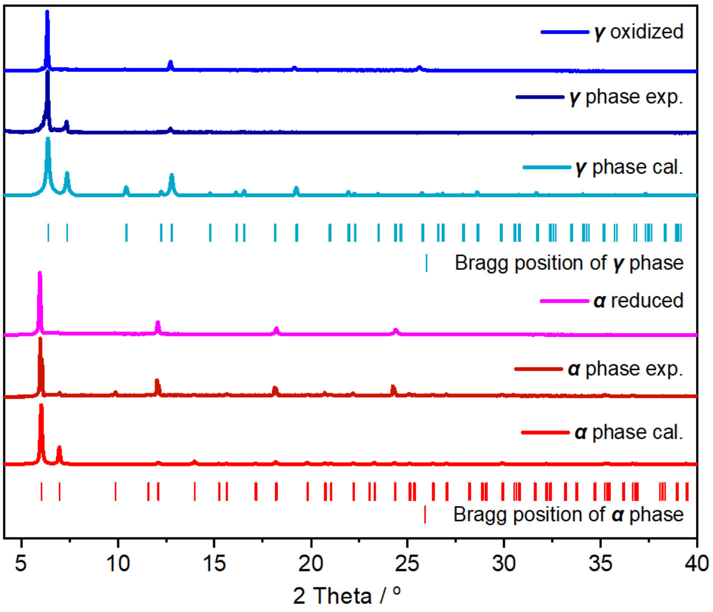

Figure S20 Comparison of PXRD patterns between calculated and experimental **fcu-4-Co(II),** as well as **fcu-4-Co(II)** obtained by reducing **fcu-4-Co(III)** with N_2H_4; comparison of PXRD patterns between the calculated **fcu-4-Co(III)**, **fcu-4-Co(III)** after cycling and **fcu-4-Co(III)** obtained by oxidizing **fcu-4-Co(III)** with *m*-CPBA.

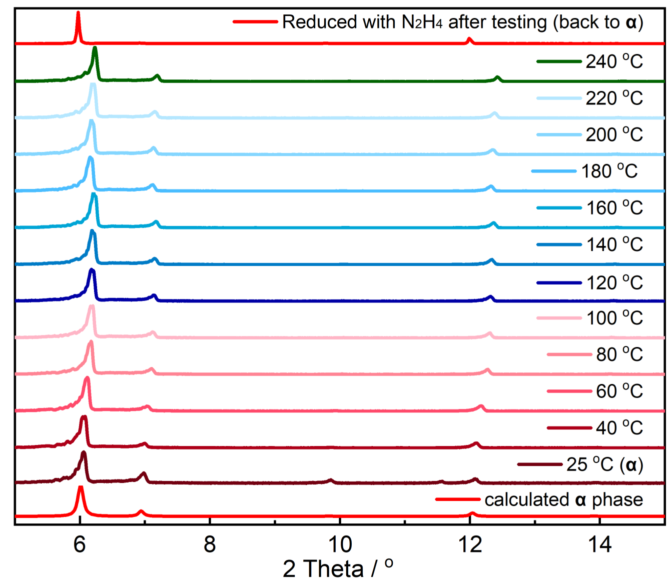

Figure S21 VT-PXRD of MeOH exchanged **fcu-1-Co(II)** under ambient air.

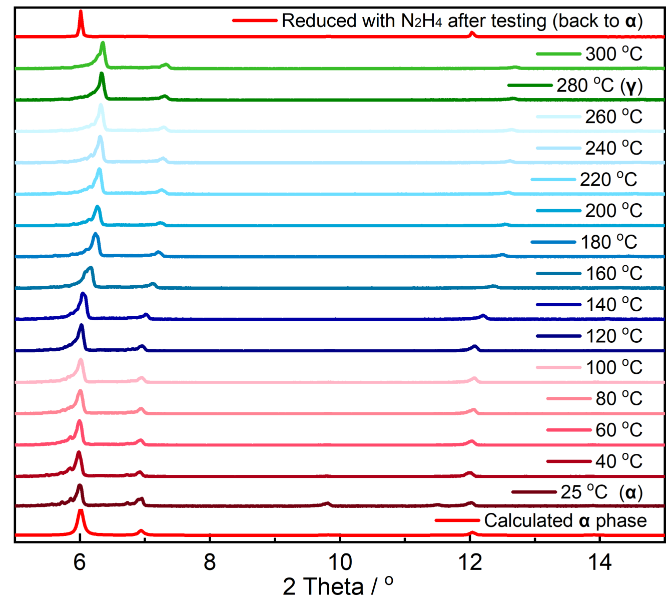

Figure S22 VT-PXRD of MeOH exchanged **fcu-1-Co(II)** under nitrogen.

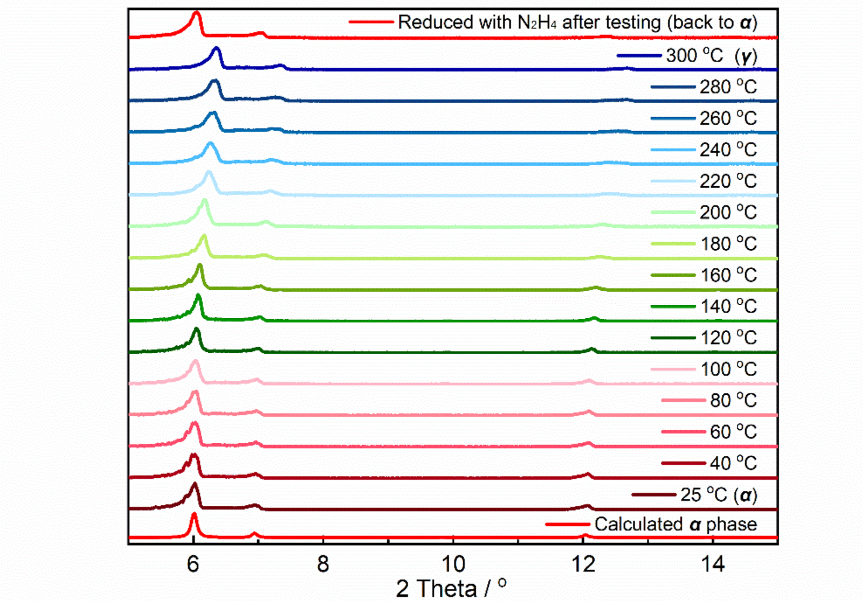

Figure S23 VT-PXRD of MeOH exchanged **fcu-1-Co(II)** under vacuum.

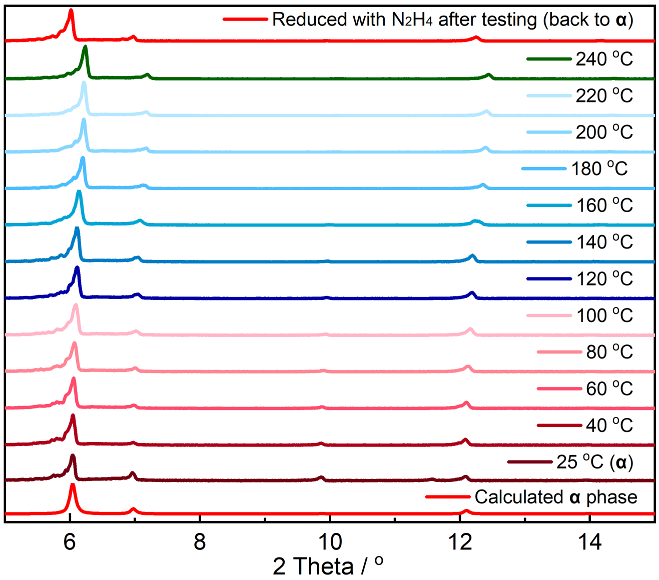

Figure S24 VT-PXRD of MeOH exchanged **fcu-2-Co(II)** under ambient air.

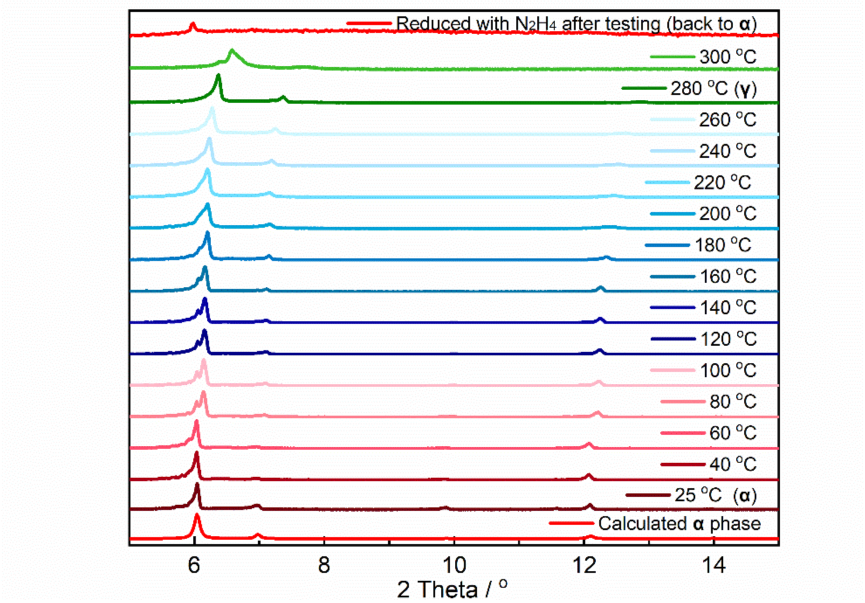

Figure S25 VT-PXRD of MeOH exchanged **fcu-2-Co(II)** under nitrogen.

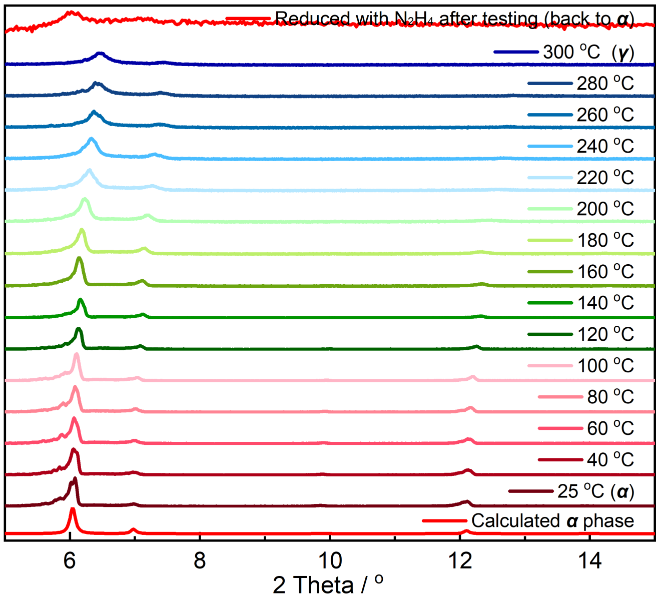

Figure S26 VT-PXRD of MeOH exchanged **fcu-2-Co(II)** under vacuum.

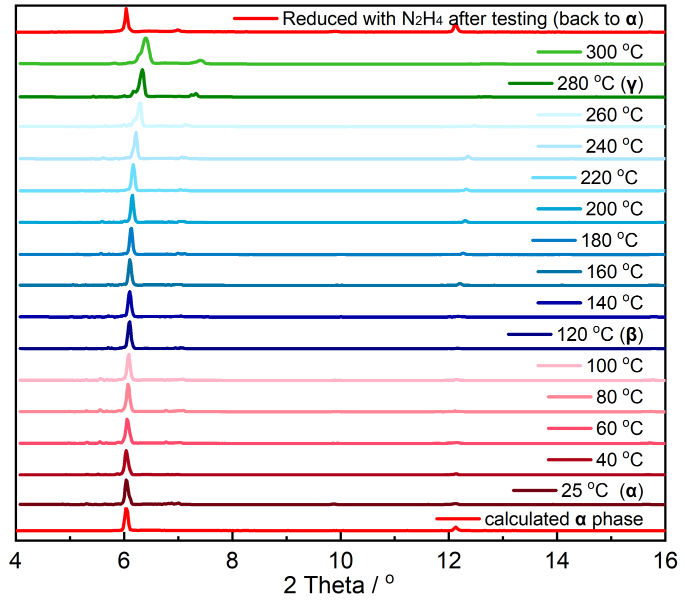

Figure S27 VT-PXRD of MeOH exchanged **fcu-3-Co(II)-α** under nitrogen.

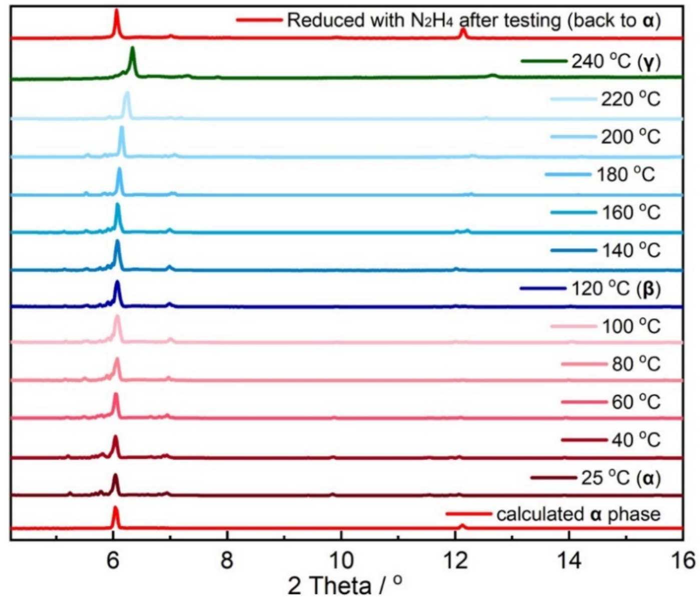

Figure S28 VT-PXRD of MeOH exchanged **fcu-3-Co(II)-α** under ambient air.

Table S3 Comparison of phase change temperature of MeOH exchanged **fcu-3-Co(II)-α** in VT-PXRD under different atmosphere.

Phase	Vacuum	N_2	Air
α	25	25	25
β	100	120	120
γ	300	280	240
Regeneration	Achieved by N_2H_4 reduction	Achieved by N_2H_4 reduction	Achieved by N_2H_4 reduction

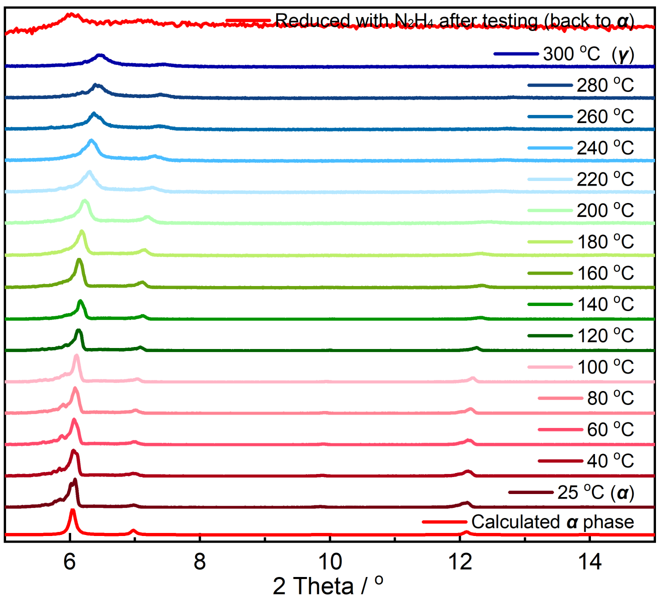

Figure S29 VT-PXRD of MeOH exchanged **fcu-4-Co(II)** under ambient air.

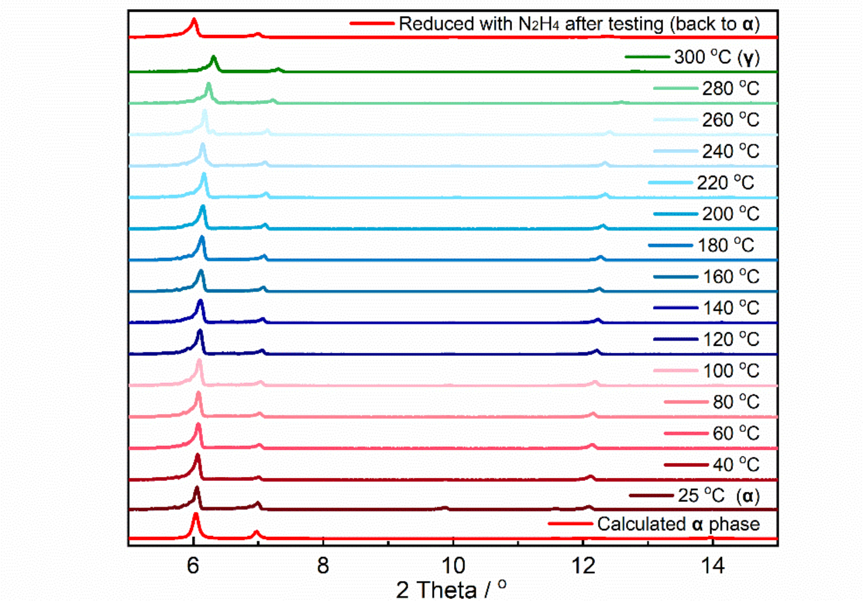

Figure S30 VT-PXRD of MeOH exchanged **fcu-4-Co(II)** under nitrogen.

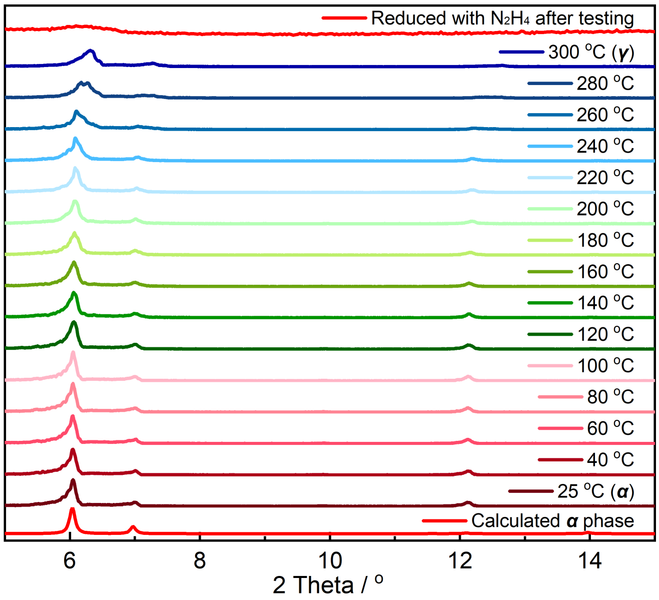

Figure S31 VT-PXRD of MeOH exchanged **fcu-4-Co(II)** under vacuum.

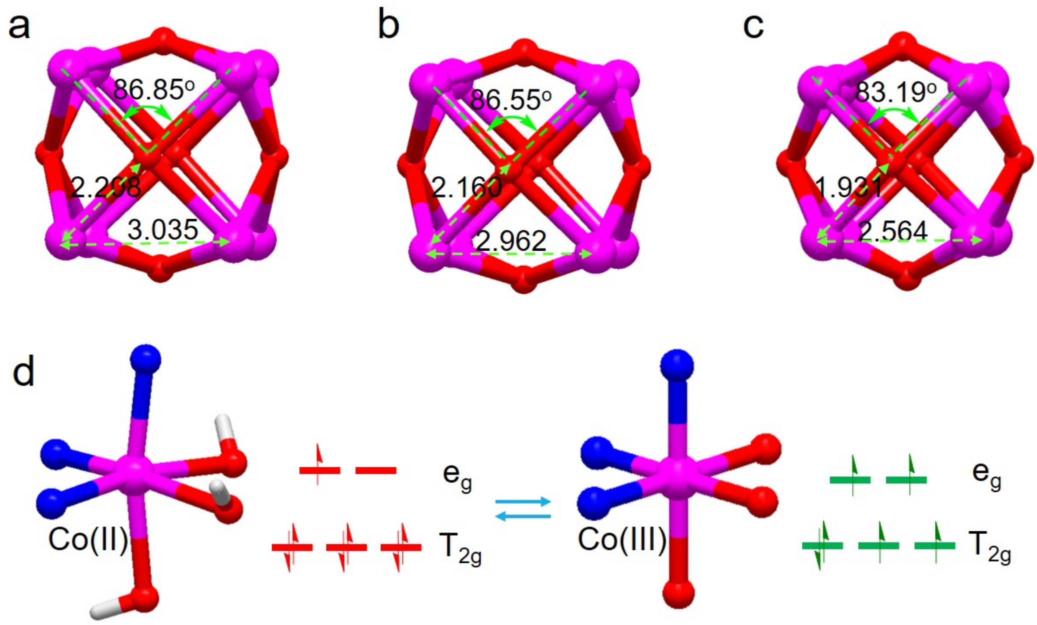

Figure S32 Structure of the Co_8 SBB in (a) **fcu-3-Co(II)-α**, (b) **fcu-3-Co(II)-β** and (c) **fcu-3-Co(III)-γ**. (d) schematic representation of the Co(II) and Co(III) possible spin states in **fcu-3-Co**.

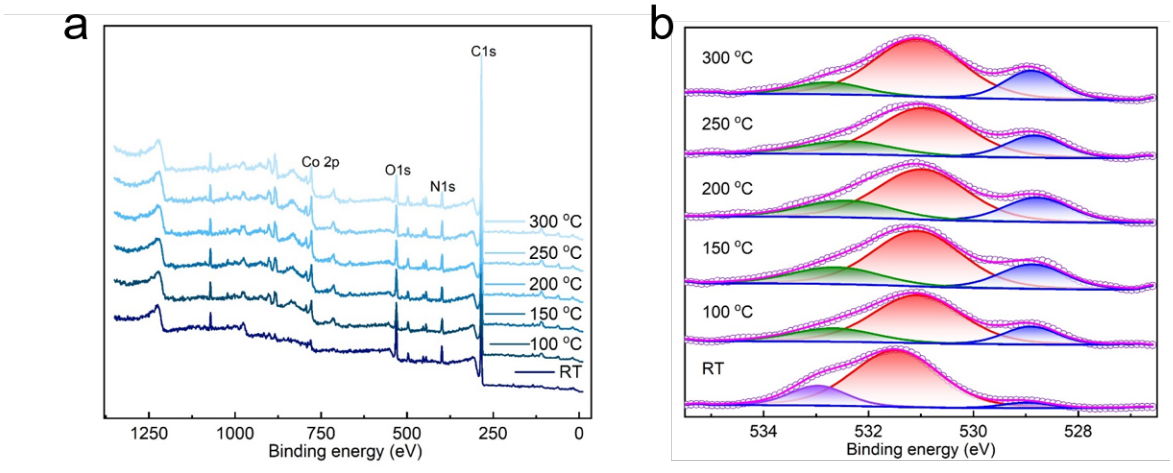

Figure S33 *In situ* XPS (a) survey and (b) O1s spectra of **fcu-3-Co(II)-*α***.

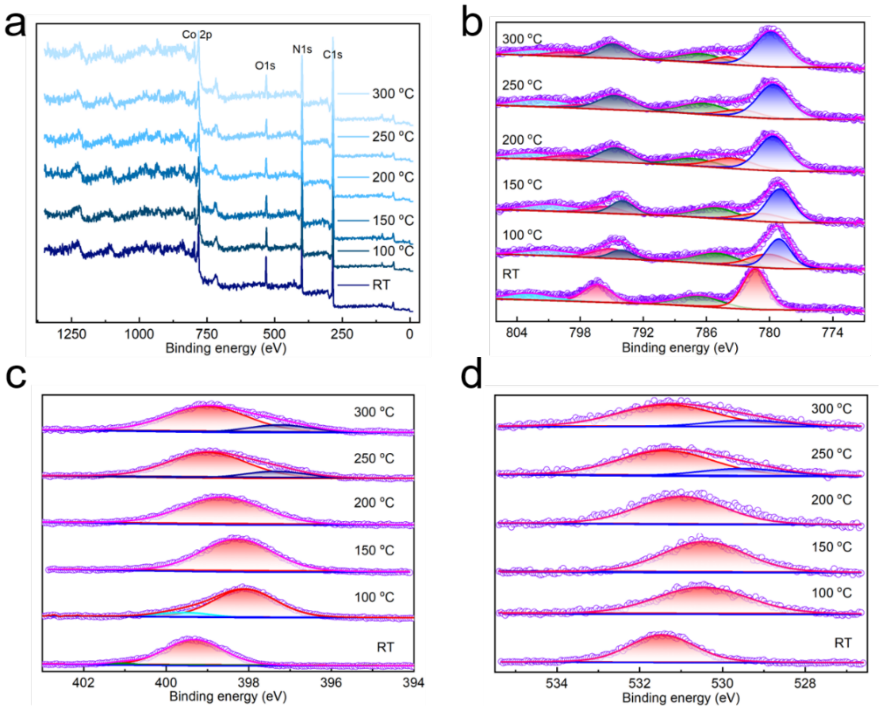

Figure S34 *In situ* XPS spectra of fcu-1-Co(II): XPS (a) survey, (b) Co 2p, (c) N 1s and (d) O1s spectra.

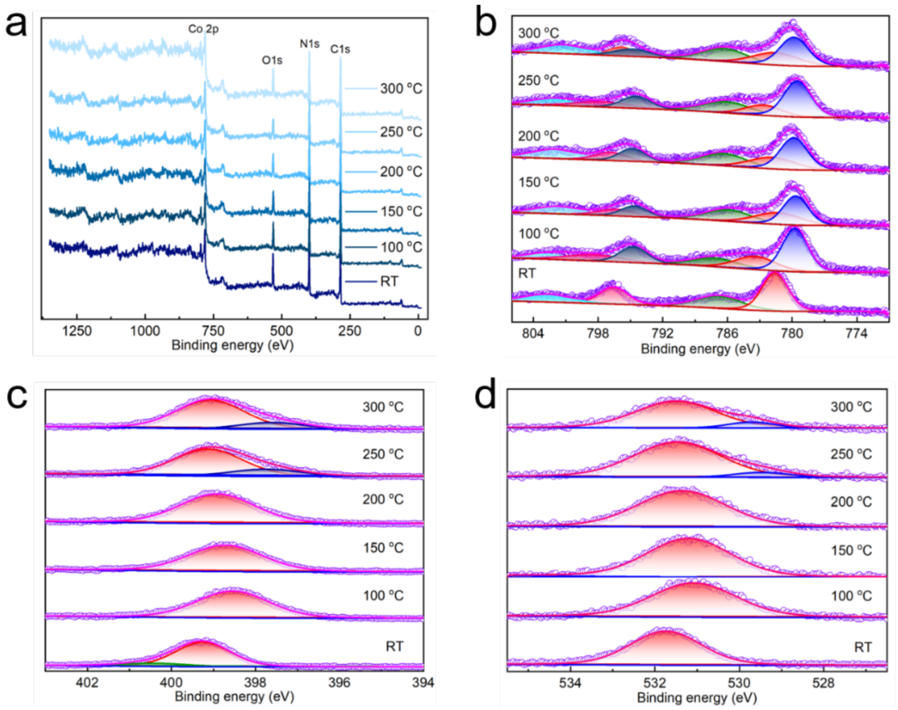

Figure S35 *In situ* XPS spectra of fcu-2-Co(II): XPS (a) survey, (b) Co 2p, (c) N 1s and (d) O1s spectra.

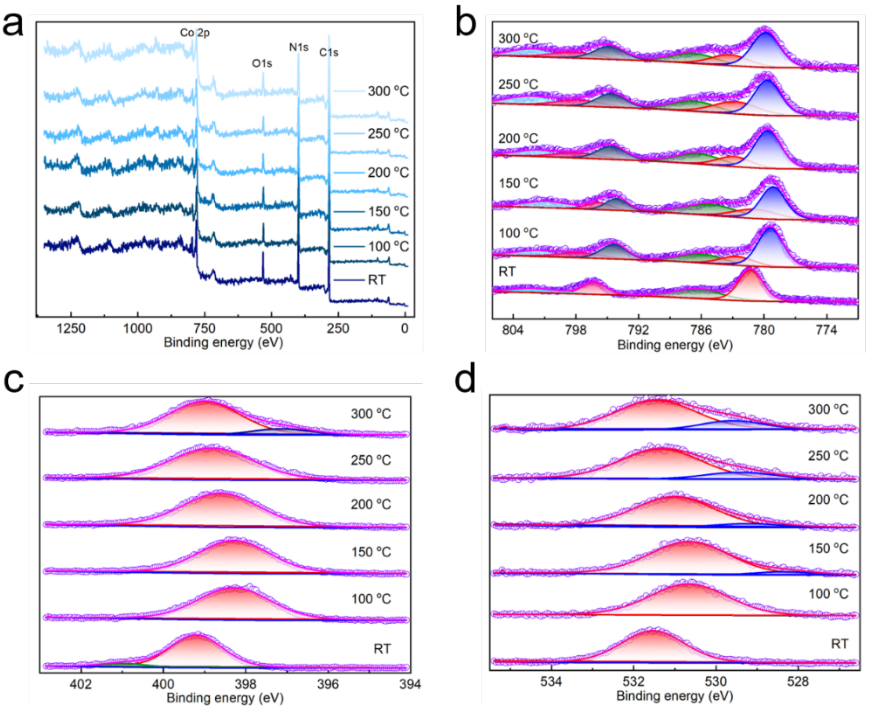

Figure S36 *In situ* XPS spectra of fcu-4-Co(II): XPS (a) survey, (b) Co 2p, (c) N 1s and (d) O1s spectra.

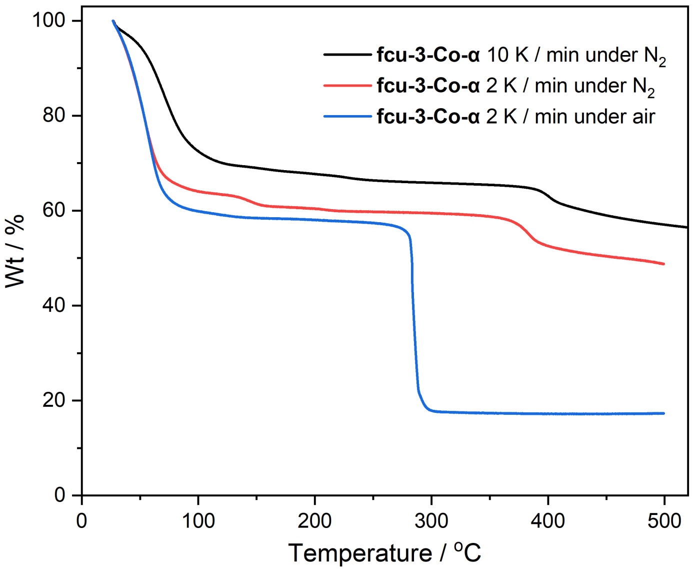

Figure S37 TGA curves of air-dried **fcu-3-Co(II)-α** after MeOH exchange with different ramping rate under different atmosphere.

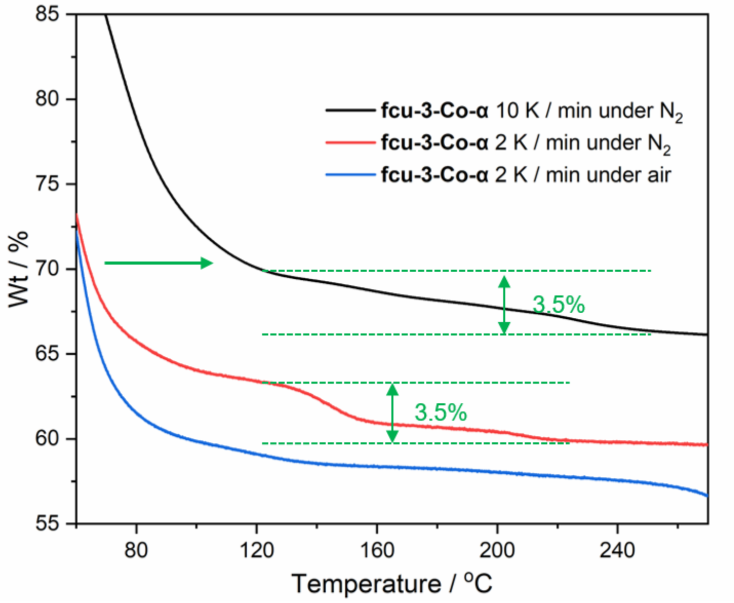

Figure S38 Zoom-in TGA curves of air-dried **fcu-3-Co(II)-α** after MeOH exchange with different ramping rate under different atmosphere. The weight loss was observed between 120-250 °C under N_2 with a temperature ramping rate of 10 K / min. When the ramping rate was 2 K / min, the weight loss was observed to complete at lower temperature. In the second round of cycling measurement (Figure S34-35), the initial mass could be recovered to zero upon re-activation at 150 °C, which might correspond to the same weight loss.

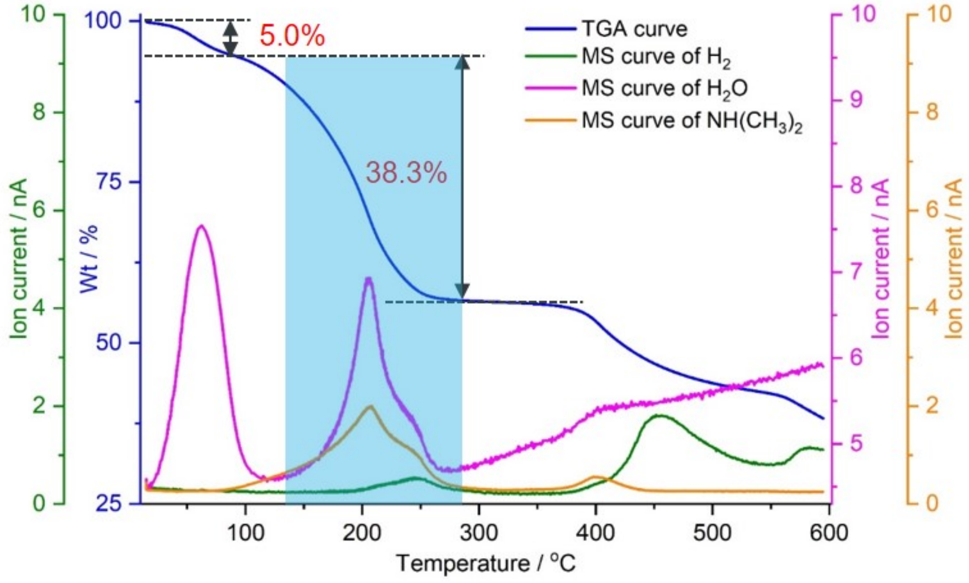

Figure S39 TGA-MS spectra of **fcu-3-Co(II)-α** filtered from mother liquid.

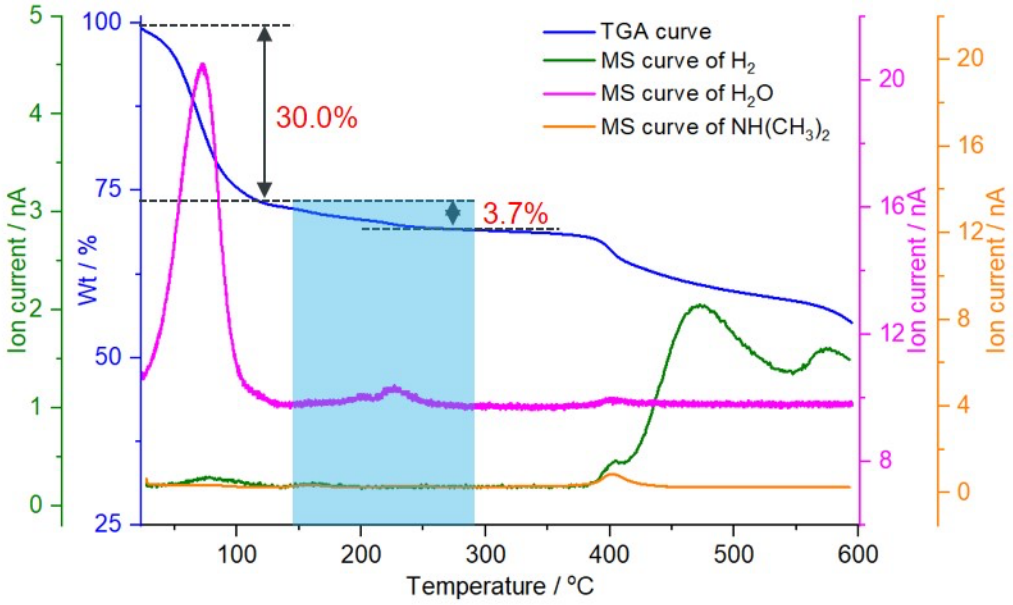

Figure S40 TGA-MS spectra of **fcu-3-Co(III)-γ**.

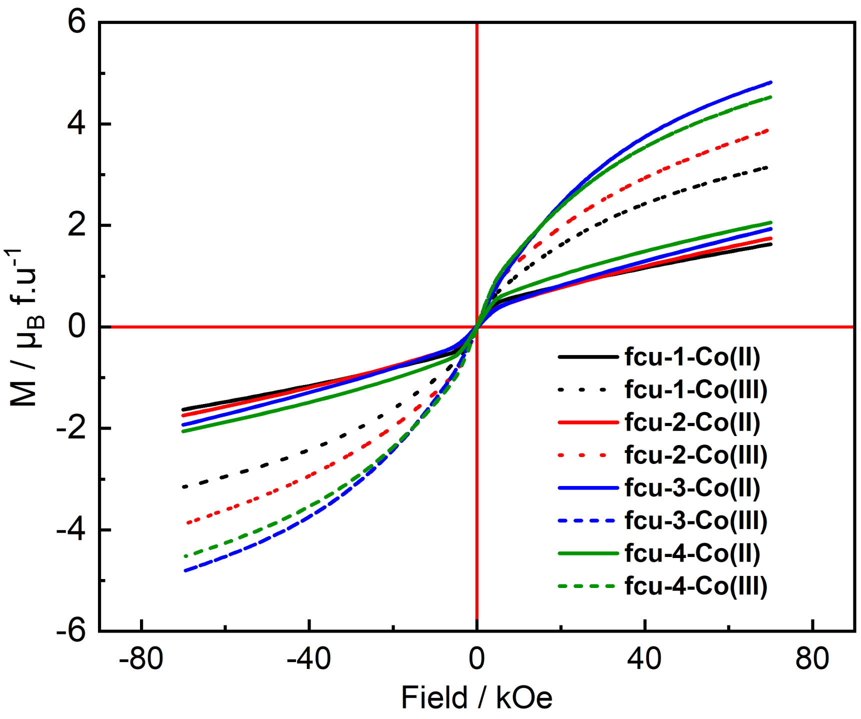

Figure S41 SQUID magnetometry moment vs field (MvH) measurements of **fcu-L-Co(II)** and four **fcu-L-Co(III)**.

Table S4 Comparison of parameters in water sorption among the lead performing adsorbents.

MOFs	Inflection point (RH / %)	Uptake (cm^3_{water}/g_{MOF}) at 30% RH	Uptake (g_{water}/g_{MOF}) at 30% RH	Capacity (g_{water}/g_{MOF}) between 5-30% RH[a]	BET surface area (m^2/g)	Comment	Ref
Co_2Cl_2(BTDD)	29	1083	0.87	0.79	2322		S9
fcu-1-Co(II)	26	684	0.55	0.44	1656		This work
fcu-2-Co(II)	22	697	0.56	0.44	1642		This work
fcu-3-Co(II)	26	846	0.68	0.60	1693		This work
fcu-4-Co(II)	25	759	0.61	0.53	1750		This work
fcu-1-Co(III)	23	386	0.31	0.27	1026		This work
fcu-2-Co(III)	20	361	0.29	0.25	1081		This work
fcu-3-Co(III)	23	560	0.45	0.41	1119		This work
fcu-4-Co(III)	20	448	0.36	0.31	1057		This work
MOF-LA2-1	27	697	0.56	0.5	1892		S10
MIP-211	29	635	0.51	0.46	1450		S11
MOF-808-FR	28	622	0.50	0.46	1610		S12
MOF-808PDS1.7-1 (the first cycle)	21	560	0.45	0.4	1365	Capacity loss in the 2nd cycle	S13
MOF-841	25	547	0.44	0.43	1390		S14
Zr-adip	12	523	0.42	0.31	1214		S15
MOF-303	13	498	0.40	0.33	1355		S16
ZrMOF-1	30	498	0.40	0.33	1877		S17
MIP-200[b]	18	485	0.39	0.29	1000		S18
MOF-333	22	473	0.38	0.36	1321		S16
MOF-808PDS1.7-2 (the second cycle)	21	473	0.38	0.34	1365		S13
MIL-125(Ti)-NH_2	20	448	0.36	0.32	1230		S19
CAU-23(Al)	27	435	0.35	0.33	1217		S20
MIL-160(Al)[b]	9	435	0.35	0.3	1070		S21
Al-fumarate	27	411	0.33	0.31	1021		S22
MFU-4	25	398	0.32	0.29	1611		S23
CAU-10(Al)-H	16	386	0.31	0.30[c]	635		S24
Zeolite 13X	1	386	0.31	0.03	650		S14
BUT-155	26	386	0.31	0.23	2070	Capacity loss in the 2nd cycle	S25
Mg-CUK-1[b]	26	373	0.30	0.29[c]	N.A.		S26
Co-CUK-1[b]	12	348	0.28	0.26	N.A.		
MOF-801	9	323	0.26	0.19	990		S27

FeFFIVE-1-Ni	2	286	0.23	0.05[c]	258		S28
AlFFIVE-1-Ni	2	274	0.22	0.02[c]	324		
ROS-39[d]	8	274	0.22	0.10	N.A.		S29
Cu-AD-SA	12	224	0.18	0.15	651		S30
ROS-40[d]	13	174	0.14	0.14	N.A.		S29
UiO-66	34	124	0.10	0.08	1290		S31
ROS-37[d]	15	100	0.08	0.08	N.A.		S29
UiO-67	54	62	0.05	0.04	2500	Capacity loss in the 2nd cycle	S31
	[a]Water sorption isotherms were collected at 298 K, and the capacity was calculated as difference between the adsorption uptake at 30% RH and desorption uptake at 5% RH; [b]Isotherms collected at 303 K; [c]Desorption data are not available, and the capacity was calculated according to the uptake difference between 30% RH and 5% RH in adsorption isotherms. N.A. represents data not available; [d]Isotherms collected at 300 K.						

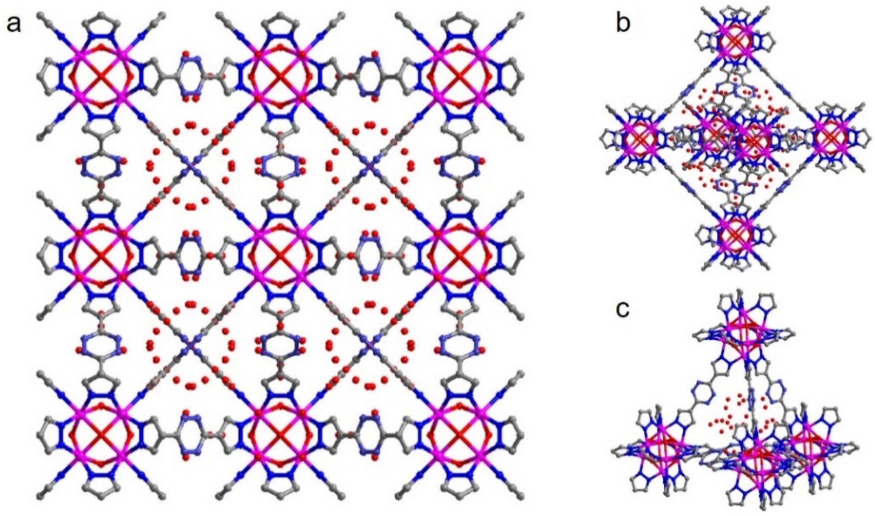

Figure S42 Structure of water-loaded **fcu-3-Co(II)-*β*** showing the location of adsorbed water molecules in (a) the framework, (b) octahedral and (c) tetrahedral cages.

Table S5 Crystallographic data and structure refinements for **fcu-3-Co(II)-H_2O**.

Name	**fcu-3-Co(II)-H_2O**	μ / mm^{-1}	7.262
Formula	$C_{60}H_{200}Co_8N_{36}O_{84}$	T / K	100.02(10)
M.W.	3240.71	Reflections collected	20392
Crystal system	Cubic	Independent reflections	879<br>R_{int} = 0.0356
Space group	$Fm\bar{3}m$	Goodness-of-fit on F^2	1.025
a/ Å	25.0833(3)	R_1[a], wR_2[b] [$I > 2\sigma(I)$]	R_1 = 0.0990<br>wR_2 = 0.2867
b/ Å	25.0833(3)		
c/ Å	25.0833(3)		
α/ °	90	R_1[a], wR_2[b] (all data)	R_1 = 0.0998<br>wR_2 = 0.2881
β/ °	90		
γ/ °	90		
V/ Å^3	15781.7(6)	Largest diff. peak and hole (e.Å^{-3})	0.75/–1.09

[a] $R_1=\Sigma||F_o|-|F_c|| / \Sigma|F_o|$

[b] $wR_2=\{\Sigma[w(F_o^2-F_c^2)^2]/[w(F_o^2)^2]\}^{1/2}$, $[F_o>4\sigma(F_o)]$

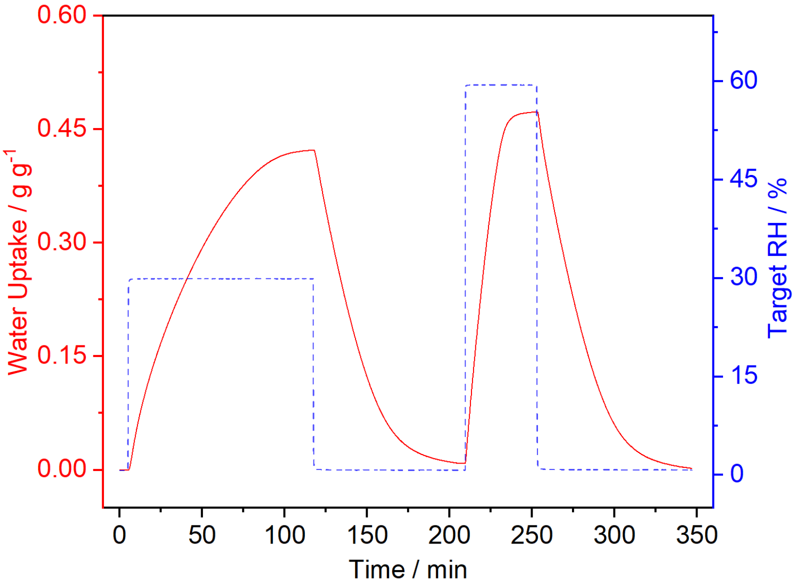

Figure S43 Kinetics curve of **fcu-1-Co(II)** for 0-30% and 0%–60%RH humidity swing.

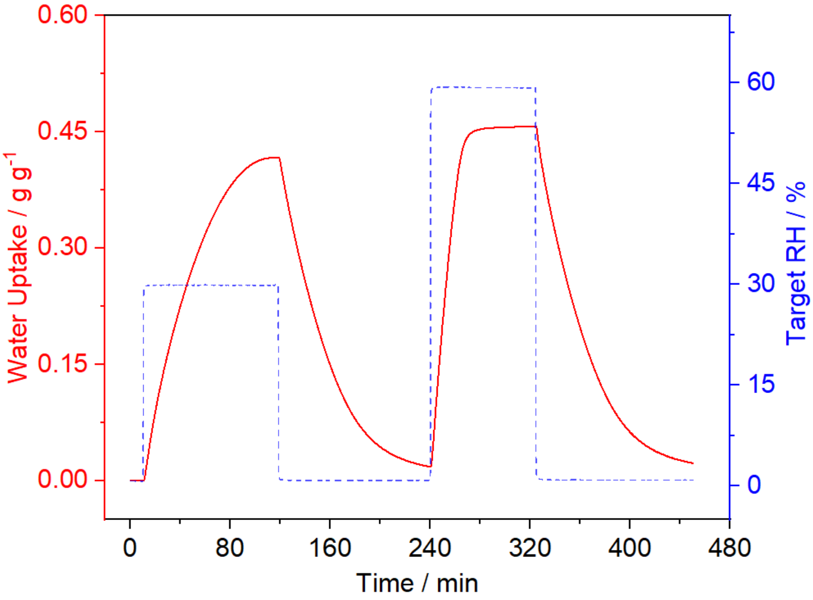

Figure S44 Kinetics curve of **fcu-2-Co(II)** for 0-30% and 0–60%RH humidity swing.

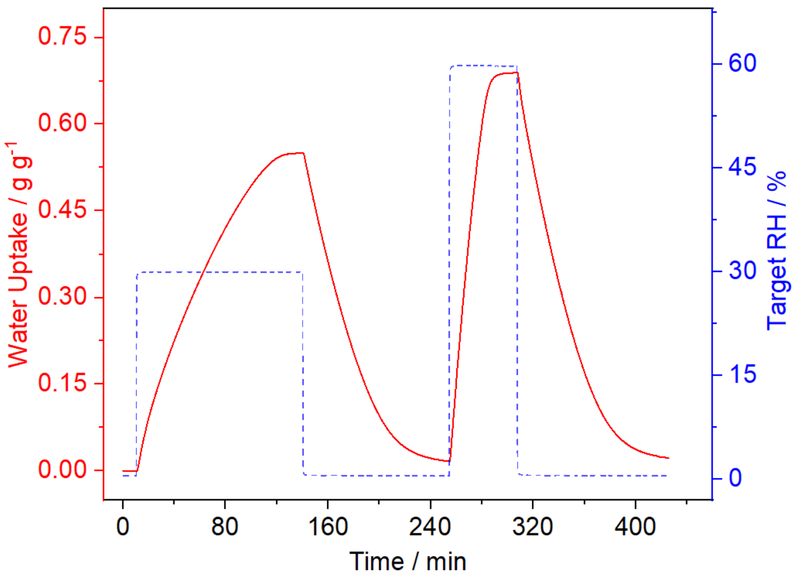

Figure S45 Kinetics curve of **fcu-3-Co(II)** for 0–30% and 0–60%RH humidity swing.

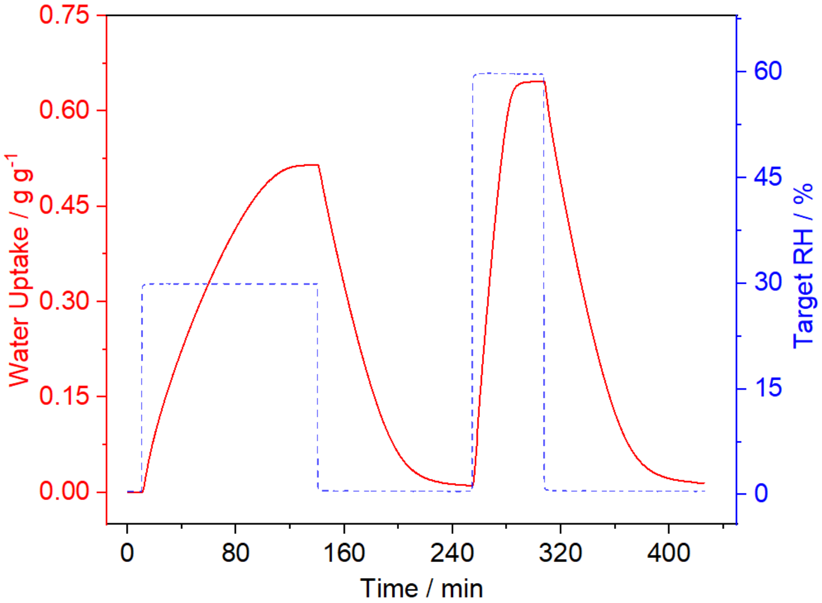

Figure S46 Kinetics curve of **fcu-4-Co(II)** for 0–30% and 0–60%RH humidity swing.

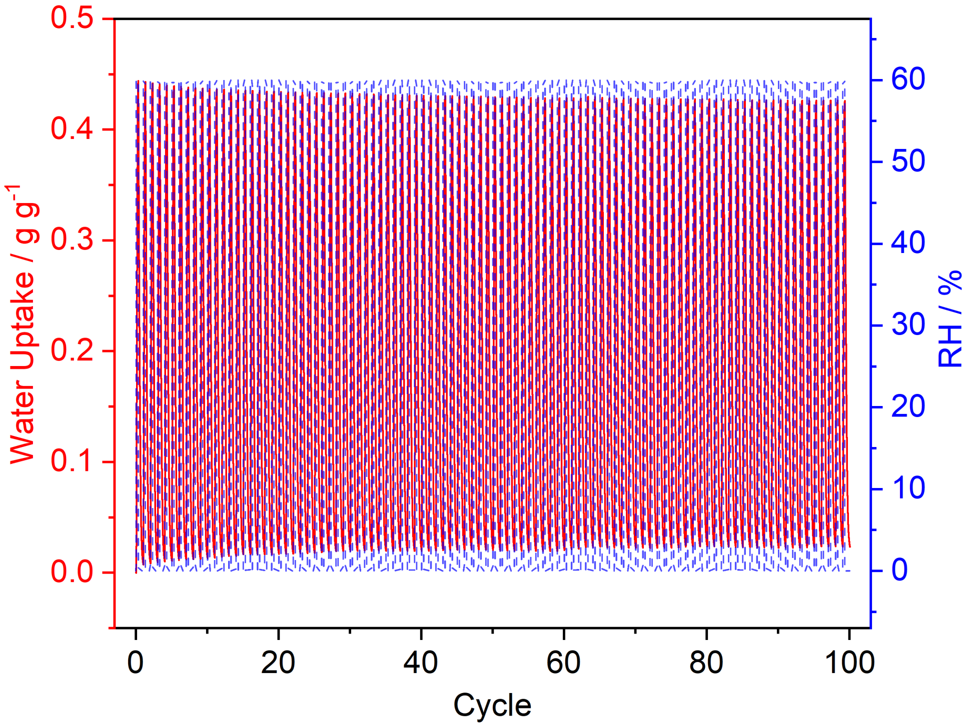

Figure S47 Water sorption cycling profile of **fcu-1-Co(II)** after the first cycle of isotherms test.

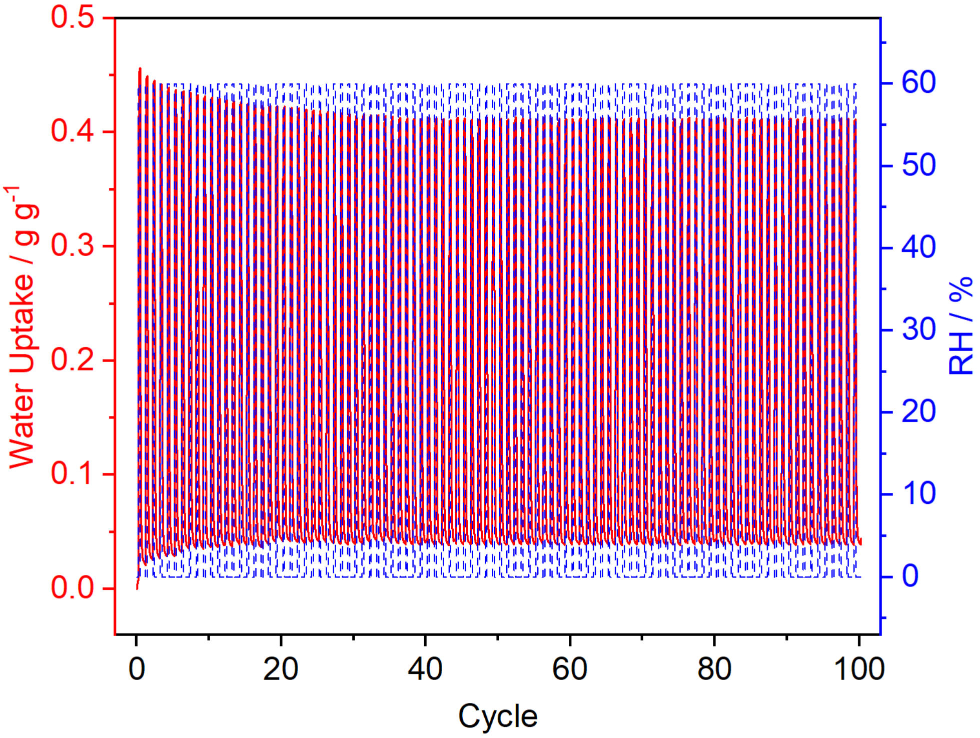

Figure S48 Water sorption cycling profile of **fcu-2-Co(II)** after the first cycle of isotherms test.

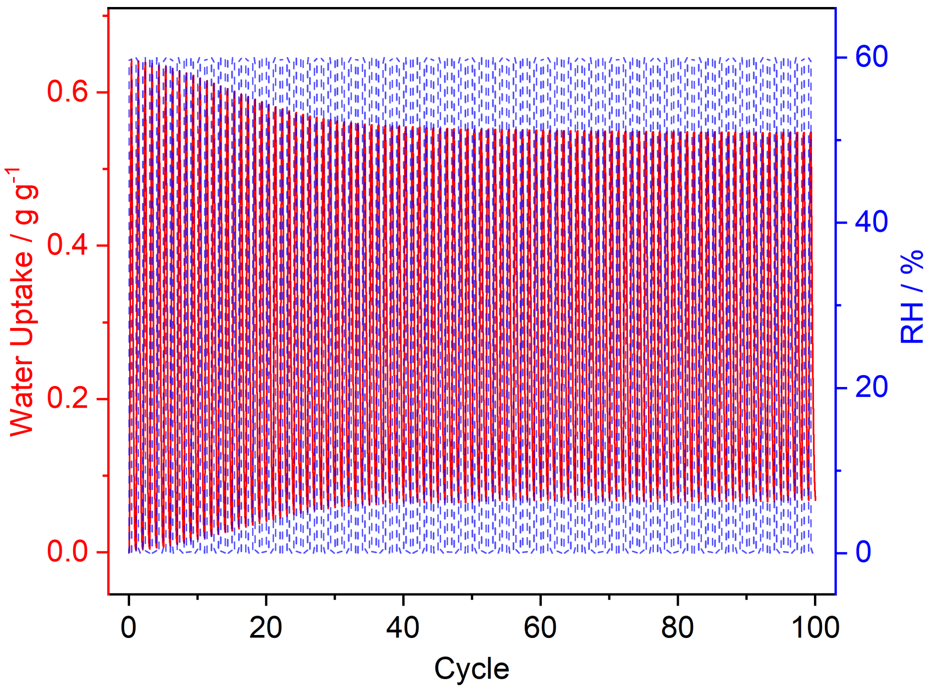

Figure S49 Water sorption cycling profile of **fcu-4-Co(II)** after the first cycle of isotherms test.

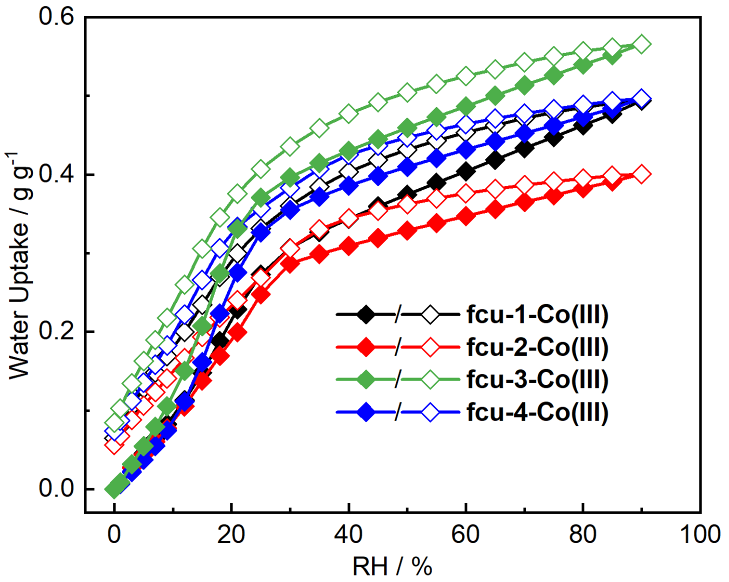

Figure S50 Water sorption isotherms of **fcu-L-Co(III)**.

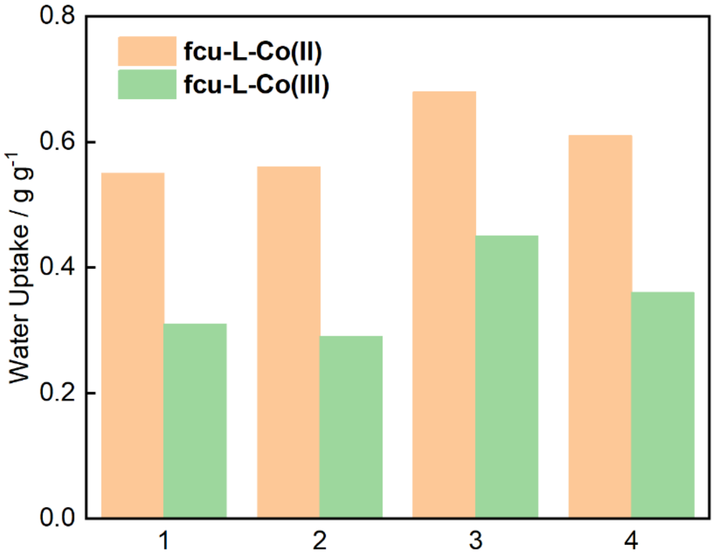

Figure S51 A bar chart comparing initial (orange) and final (green) water vapor sorption capacities on **fcu-L-Co**.

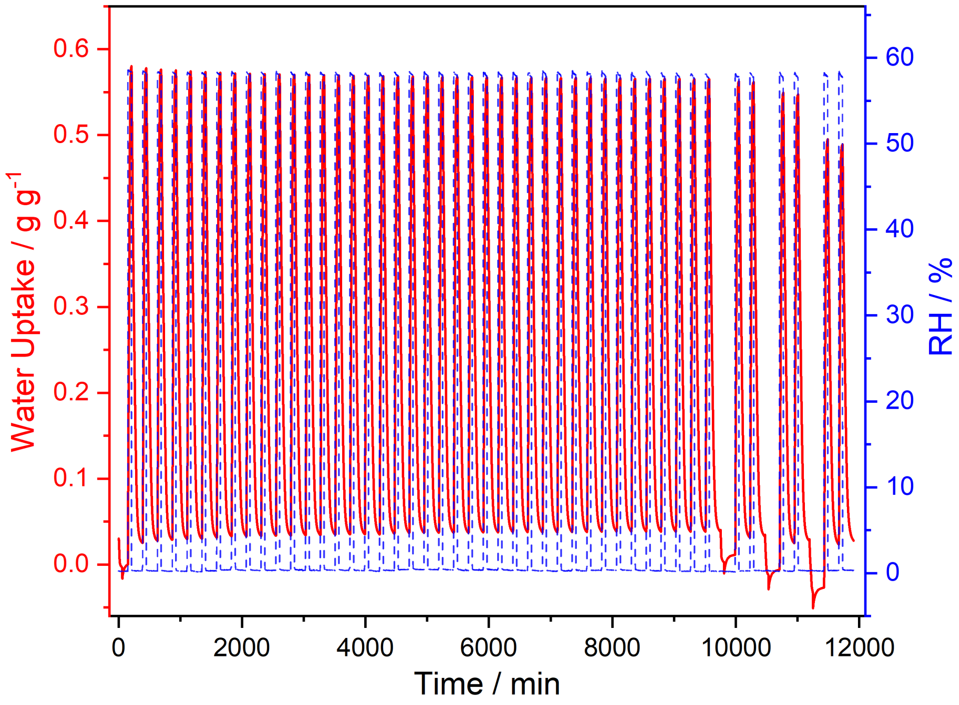

Figure S52 Second round programmed water sorption cycling profile of **fcu-3-Co(III)**.

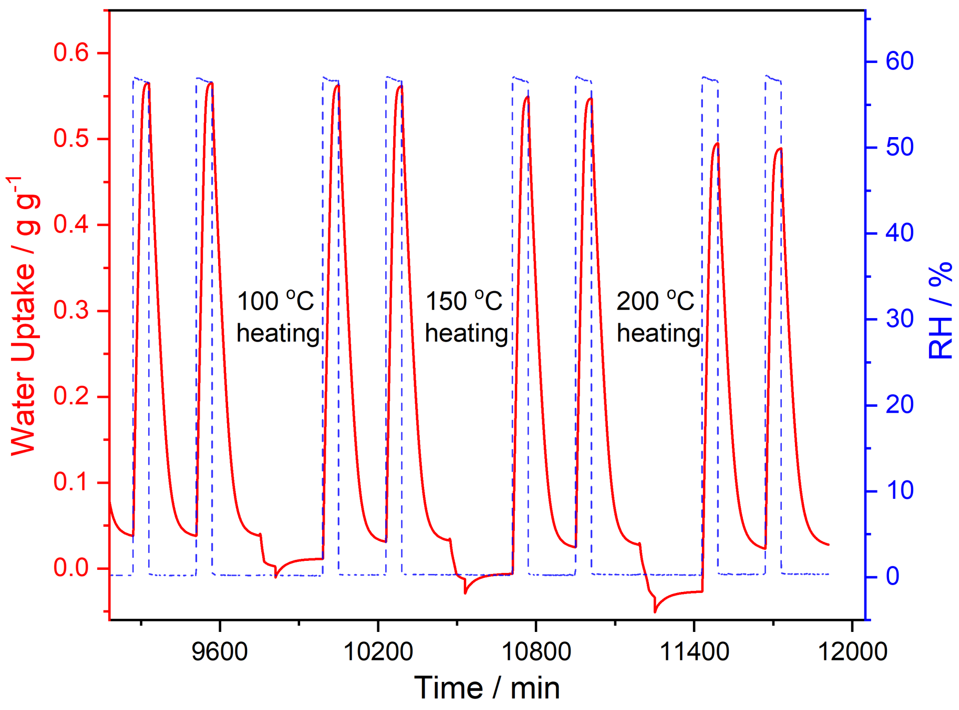

Figure S53 Zoom-in view of water sorption cycling profile of **fcu-3-Co(III)** after 40 cycles and heating.

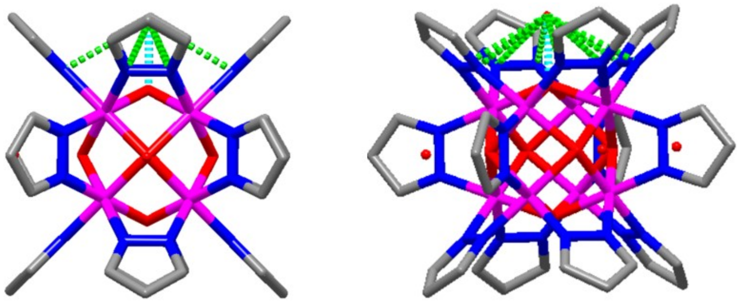

Figure S54 Structure of the Co_8 SBB with H_2O entities in **fcu-3-Co(III)**.

Section 10. References

[S1] He, T.; Huang, Z.; Yuan, S.; Lv, X.-L.; Kong, X.-J.; Zou, X.; Zhou, H.-C.; Li, J.-R. Kinetically Controlled Reticular Assembly of a Chemically Stable Mesoporous Ni(II)-Pyrazolate Metal–Organic Framework. *J. Am. Chem. Soc.* **2020**, *142*, 13491.

[S2] Kumar, A.; Hua, C.; Madden, D. G.; O'Nolan, D.; Chen, K.-J.; Keane, L.-A. J.; Perry, J. J.; Zaworotko, M. J., Hybrid ultramicroporous materials (HUMs) with enhanced stability and trace carbon capture performance. *Chem.Commun.* **2017**, *53*, 5946-5949.

[S3] Krause, L.; Herbst-Irmer, R.; Sheldrick, G. M.; Stalke, D., Comparison of silver and molybdenum microfocus X-ray sources for single-crystal structure determination. *J. Appl. Crystallogr.* **2015**, *48*, 3-10.

[S4] Shen, J.; He, X.; Ke, T.; Krishna, R.; van Baten, J. M.; Chen, R.; Bao, Z.; Xing, H.; Dincă , M.; Zhang, Z.; Yang, Q.; Ren, Q., Simultaneous interlayer and intralayer space control in two-dimensional metal−organic frameworks for acetylene/ethylene separation. *Nat. Commun.* **2020**, *11*, 6259.

[S5] Oxford Diffraction. CrysAlis Pro Software, Ver. 1.171.34 (Oxford Diffraction Ltd), **2010**.

[S6] Sheldrick, G. M. A short history of SHELX. *Acta Crystallogr. A* **2008**, *64*, 112.

[S7] Spek, A.L. Structure validation in chemical crystallography. *Acta Crystallogr. D Biol. Crystallogr.* **2009**, *65*, 148.

[S8] Rees, B.; Jenner, L.; Yusupov, M. Acta Crystallogr. Sect. D, Biological Crystallography, **2005**, *61*, 1299.

[S9] Rieth, A. J.; Yang, S.; Wang, E. N.; Dinca, M. *ACS Cent. Sci.* **2017**, *3*, 668-672.

[S10] Hanikel, N.; Kurandina, D.; Chheda, S.; Zheng, Z.; Rong, Z.; Neumann, S. E.; Sauer, J.; Siepmann, J. I.; Gagliardi, L.; Yaghi, O. M. *ACS Cent. Sci.* **2023**, *9*, 551-557.

[S11] Matemb Ma Ntep, T. J.; Wahiduzzaman, M.; Laurenz, E.; Cornu, I.; Mouchaham, G.; Dovgaliuk, I.; Nandi, S.; Knop, K.; Jansen, C.; Nouar, F.; et al. *Adv. Mater.* **2023**, e2211302.

[S12] Chen, O. I.; Liu, C.-H.; Wang, K.; Borrego-Marin, E.; Li, H.; Alawadhi, A. H.; Navarro, J. A. R.; Yaghi, O. M. *J. Am. Chem. Soc.* **2024**, *146*, 2835–2844.

[S13] Luo, T. Y.; Park, S.; Chen, T. H.; Prerna; Patel, R.; Li, X.; Ilja Siepmann, J.; Caratzoulas, S.; Xia, Z.; Tsapatsis, M. *Angew. Chem. Int. Ed.* **2022**, *61*, 202209034.

[S14] Furukawa, H.; Gandara, F.; Zhang, Y. B.; Jiang, J.; Queen, W. L.; Hudson, M. R.; Yaghi, O. M. *J. Am. Chem. Soc.* **2014**, *136*, 4369-4381.

[S15] Li, B.; Lu, F. F.; Gu, X. W.; Shao, K.; Wu, E.; Qian, G. *Adv. Sci.* **2022**, *9*, 2105556.

[S16] Hanikel, N.; Pei, X.; Chheda, S.; Lyu, H.; Jeong, W.; Sauer, J.; Gagliardi, L.;

Yaghi, O. M. *Science* **2021**, *374*, 454-459.
[S17] Liu, W.; Wu, E.; Yu, B.; Liu, Z.; Wang, K.; Qi, D.; Li, B.; Jiang, J. *Angew. Chem. Int. Ed.* **2023**, 202305144.
[S18] Wang, S.; Lee, J. S.; Wahiduzzaman, M.; Park, J.; Muschi, M.; Martineau-Corcos, C.; Tissot, A.; Cho, K. H.; Marrot, J.; Shepard, W.; et al. *Nat. Energy* **2018**, *3*, 985-993.
[S19] Canivet, J.; Bonnefoy, J.; Daniel, C.; Legrand, A.; Coasne, B.; Farrusseng, D. *New J. Chem.* **2014**, *38*, 3102-3111.
[S20] Zheng, Z.; Hanikel, N.; Lyu, H.; Yaghi, O. M. *J. Am. Chem. Soc.* **2022**, *144*, 22669-22675.
[S21] Cadiau, A.; Lee, J. S.; Damasceno Borges, D.; Fabry, P.; Devic, T.; Wharmby, M. T.; Martineau, C.; Foucher, D.; Taulelle, F.; Jun, C. H.; et al. *Adv. Mater.* **2015**, *27*, 4775-4780.
[S22] Jeremias, F.; Fröhlich, D.; Janiak, C.; Henninger, S. K. *RSC Adv.* **2014**, *4*, 24073-24082.
[S23] Biswas, S.; Grzywa, M.; Nayek, H. P.; Dehnen, S.; Senkovska, I.; Kaskel, S.; Volkmer, D. *Dalton. Trans.* **2009**, 6487-6495.
[S24] Reinsch, H.; van der Veen, M. A.; Gil, B.; Marszalek, B.; Verbiest, T.; de Vos, D.; Stock, N. *Chem. Mater.* **2012**, *25*, 17-26.
[S25] Chen, Y.; Wang, B.; Wang, X.; Xie, L. H.; Li, J.; Xie, Y.; Li, J. R. *ACS Appl. Mater. Interfaces* **2017**, *9*, 27027-27035.
[S26] Lee, J. S.; Yoon, J. W.; Mileo, P. G. M.; Cho, K. H.; Park, J.; Kim, K.; Kim, H.; de Lange, M. F.; Kapteijn, F.; Maurin, G.; et al. *ACS Appl. Mater. Interfaces* **2019**, *11*, 25778-25789.
[S27] Kim, H.; Yang, S.; Rao, S. R.; Narayanan, S.; Kapustin, E. A.; Furukawa, H.; Umans, A. S.; Yaghi, O. M.; Wang, E. N. *Science* **2017**, *356*, 430-434.
[S28] Cadiau, A.; Belmabkhout, Y.; Adil, K.; Bhatt, P. M.; Pillai, R. S.; Shkurenko, A.; Martineau-Corcos, C.; Maurin, G.; Eddaoudi, M. *Science* **2017**, *356*, 731-735.
[S29] Bezrukov, A. A.; O'Hearn, D. J.; Gascón-Pérez, V.; Darwish, S.; Kumar, A.; Sanda, S.; Kumar, N.; Francis, K.; Zaworotko, M. J. *Cell Reports Physical Science* **2023**, *4*, 101252.
[S30] Wang, L.; Wang, K.; An, H. T.; Huang, H.; Xie, L. H.; Li, J. R. *ACS Appl. Mater. Interfaces* **2021**, *13*, 49509-49518.
[S31] Lawrence, M. C.; Katz, M. J. *J. Phys. Chem. C* **2021**, *126*, 1107-1114.
